# H3K36 dimethylation by MMSET promotes classical non-homologous end-joining at unprotected telomeres

**DOI:** 10.1038/s41388-020-1334-0

**Published:** 2020-05-29

**Authors:** Inge de Krijger, Jaco van der Torre, Marieke H. Peuscher, Mathias Eder, Jacqueline J. L. Jacobs

**Affiliations:** Division of Oncogenomics, The Netherlands Cancer Institute, Plesmanlaan 121, 1066 CX Amsterdam, The Netherlands

**Keywords:** Oncogenes, Chromosomes, Histone post-translational modifications, Double-strand DNA breaks, Non-homologous-end joining

## Abstract

The epigenetic environment plays an important role in DNA damage recognition and repair, both at DNA double-strand breaks and at deprotected telomeres. To increase understanding on how DNA damage responses (DDR) at deprotected telomeres are regulated by modification and remodeling of telomeric chromatin we screened 38 methyltransferases for their ability to promote telomere dysfunction-induced genomic instability. As top hit we identified MMSET, a histone methyltransferase (HMT) causally linked to multiple myeloma and Wolf-Hirschhorn syndrome. We show that MMSET promotes non-homologous end-joining (NHEJ) at deprotected telomeres through Ligase4-dependent classical NHEJ, and does not contribute to Ligase3-dependent alternative NHEJ. Moreover, we show that this is dependent on the catalytic activity of MMSET, enabled by its SET-domain. Indeed, in absence of MMSET H3K36-dimethylation (H3K36me2) decreases, both globally and at subtelomeric regions. Interestingly, the level of MMSET-dependent H3K36me2 directly correlates with NHEJ-efficiency. We show that MMSET depletion does not impact on recognition of deprotected telomeres by the DDR-machinery or on subsequent recruitment of DDR-factors acting upstream or at the level of DNA repair pathway choice. Our data are most consistent with an important role for H3K36me2 in more downstream steps of the DNA repair process. Moreover, we find additional H3K36me2-specific HMTs to contribute to NHEJ at deprotected telomeres, further emphasizing the importance of H3K36me2 in DNA repair.

## Introduction

Efficient recognition and repair of DNA double-strand breaks (DSBs) are essential in maintaining genome integrity. Both occur in the context of the surrounding chromatin and are associated with specific chromatin alterations, including phosphorylation, ubiquitylation, methylation, and acetylation of histones that control recruitment of DNA damage responses (DDR)-proteins [1]. This is exemplified by 53BP1 binding to both dimethylated H4K20 (H4K20me2) and damage-induced H2AK15-ubiquitin to promote non-homologous end-joining (NHEJ)-mediated DNA repair [2, 3] and its counteraction by histone acetylation to shift toward repair by homologous recombination (HR) [4–6]. Finally, changes in chromatin compaction facilitate DSB repair, and different chromatin states impact on repair-efficiency, as seen by differences in repair-speed between heterochromatin and euchromatin [1, 7, 8].

Telomeres are complex nucleoprotein structures that cap natural chromosome ends to protect them from being inadvertently recognized and processed as damaged DNA, thereby maintaining genome stability [9, 10]. Telomeres are repetitive in nature and resemble heterochromatin by containing HP1, H3K9me3, H4K20me3, and low levels of H3- and H4-acetylation [11, 12]. These marks are dynamic, as telomere repeat shortening decreases the repressive H3K9me3 and H4K20me3 marks and increases histone acetylation [13]. Telomere dysfunction due to extensive shortening of telomeric TTAGGG-repeat DNA, or functional loss of the telomere-specific protein complex shelterin, results in recognition of natural chromosome ends as DSBs. This triggers a damage response resembling the DDR at DNA DSBs [14]. It is initiated by MRN (MRE11/RAD50/NBS1) and ATM and ATR kinases that by activating a DNA damage checkpoint force cells into apoptosis or senescence. Simultaneously, ATM-induced DDR signaling at uncapped telomeres recruits repair factors that through NHEJ cause chromosome end-to-end fusions [12, 15]. Also in telomere dysfunction chromatin plays important roles. For instance, inhibiting RNF8-mediated chromatin ubiquitylation, inhibiting the chromatin remodeler CHD2, or decreasing the heterochromatic state with HDAC-inhibitors or SUV39H1/2 depletion, impairs NHEJ at telomeres [16–19]. Also, depletion of Ring1b, described to affect chromatin compaction, impairs telomere fusion [16].

Here, we identified the SET-domain containing histone methyltransferase (HMT) MMSET (multiple myeloma SET domain, a.k.a NSD2 or WHSC1), as a factor contributing to telomere-induced genomic instability. *MMSET* is deleted in human Wolf-Hirschhorn syndrome and dysregulated in multiple myeloma patients with a t(4;14) translocation, in which the translocation-dependent overexpression of MMSET drives oncogenic transformation [20–25]. Moreover, *MMSET* mRNA and protein levels are increased in multiple cancers [26, 27]. Interestingly, MMSET has been implicated in the repair of DNA lesions caused by various DNA-damaging sources [28–30]. Here, we describe a novel role for MMSET in controlling DNA repair at telomeres. We find that MMSET promotes Ligase4-dependent c-NHEJ at uncapped telomeres and thereby genomic instability, in a manner directly correlating with its ability to catalyze H3K36-dimethylation (H3K36me2). Since upstream control of NHEJ by ATM-signaling and 53BP1-mediated inhibition of DNA end-resection were unaffected by MMSET depletion, we hypothesize that MMSET, through catalyzing H3K36me2, affects the engagement or activity of factors acting downstream in NHEJ. Furthermore, we identified additional H3K36-methyltransferases that contribute to telomere-NHEJ. Altogether, this suggests an important role for H3K36me2 in the processing of dysfunctional telomeres.

## Results

### MMSET regulates telomere dysfunction-induced genomic instability

**Fig. 1:**
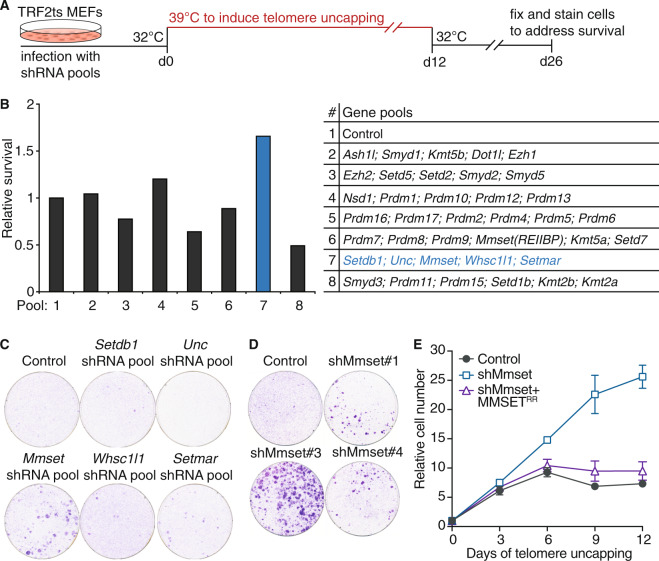
MMSET identified as a novel telomere-induced genomic instability regulator. **a** Experimental setup of the survival screen shown in (**b**). After infection with the retroviral shRNA-pools, cells were grown at the nonpermissive temperature (39 °C) to induce telomere uncapping for 12 days and returned to 32 °C for 14 days prior to staining with crystal violet. **b** Relative survival of TRF2ts MEFs infected with the indicated shRNA target gene pools (*Unc* = uncharacterized). After staining, crystal violet was extracted for quantification. Survival in control infected cells was used as reference (=1). **c** Survival assay in TRF2ts MEFs with deconvoluted shRNA-pool 7 used in (**b**). **d** Survival assay in TRF2ts MEFs infected with individual shRNAs targeting *Mmset*. **e** Growth curves of control or MMSET-depleted TRF2ts MEFs complemented with control or shRNA-resistant (RR) Flag-tagged MMSET^RR^ and grown at 39 °C for the indicated days (data represent mean ± s.d. of a technical triplicate).

To better understand how modification of chromatin affects recognition and processing of uncapped telomeres we set out to identify histone modifying enzymes that contribute to telomere-induced genomic instability. For this we used *Trf2*^−/−^;*p53*^−/−^ mouse embryo fibroblasts (MEFs) containing a temperature-sensitive allele of the shelterin component TRF2 (TRF2^Ile468Ala^; TRF2ts) [31]. Culturing TRF2ts MEFs at nonpermissive temperatures (37–39 °C) causes TRF2 to dissociate from telomeres and recognition of telomeres as DNA DSBs. This results in chromosome end-to-end fusions and severe chromosomal instability, causing cells to die of crisis or enter an irreversible growth arrest. Inhibiting factors critical in the end-joining of deprotected telomeres enables these cells to survive despite prolonged telomere uncapping, as we previously showed for Ligase4, RNF8, RNF168, and MAD2L2 [18, 32]. We selected 38 known or predicted methyltransferases and inhibited their expression using a short hairpin RNA (shRNA) library. TRF2ts MEFs were transduced with the library, cultured for 12 days at the nonpermissive temperature (39 °C) to induce telomere uncapping and returned to 32 °C to promote expansion of surviving cells (Fig. 1a). Interestingly, one shRNA-pool (pool 7) clearly rescued cell viability after prolonged telomere uncapping (Fig. 1b). Deconvolution of this shRNA-pool into single target gene pools identified the HMT *Mmset* as being responsible for the observed survival (Fig. 1c). Multiple independent shRNAs targeting *Mmset* rescued telomere dysfunction-induced lethality to an extent correlating with MMSET levels (Fig. 1d, Supplementary Fig. 1A). Indeed, cells depleted of MMSET continued proliferating despite telomere uncapping (Fig. 1e). Moreover, complementation of MMSET-depleted cells with expression of full-length MMSET cDNA abolished the rescue of cell proliferation in conditions of telomere uncapping (Fig. 1e, Supplementary Fig. 1B, C), showing that this effect is specific for MMSET. Importantly, *Mmset* knockdown did not affect cell cycle distribution (Supplementary Fig. 1D, E), excluding disturbed cell cycle kinetics as potential factor in escape from genomic crisis. Together, these results identify MMSET as a novel regulator of telomere dysfunction-induced genomic instability.

### MMSET facilitates c-NHEJ at uncapped telomeres

**Fig. 2:**
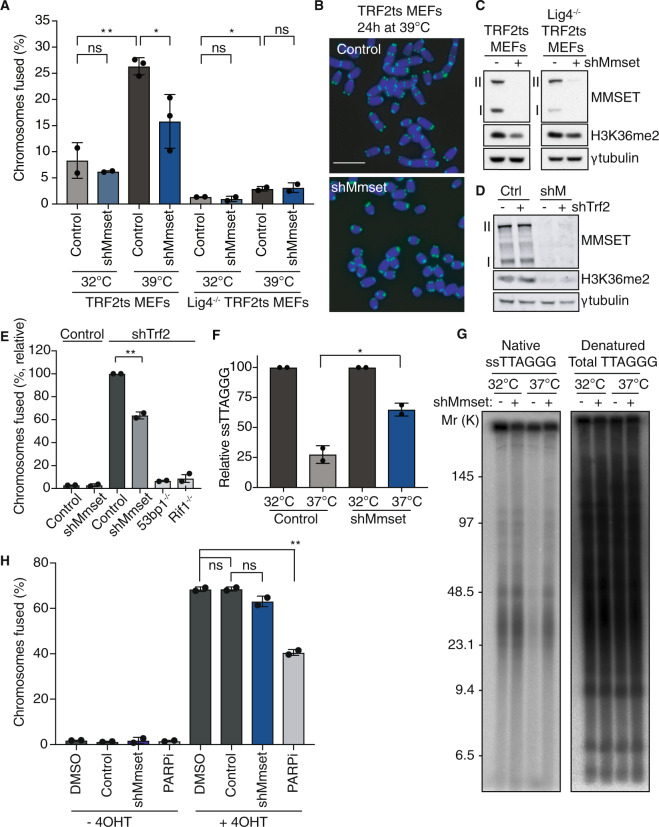
MMSET induces NHEJ-mediated telomere fusion and G-overhang degradation. **a** Chromosome fusions in TRF2ts MEFs and LigIV^−/−^ TRF2ts MEFs transduced with control or *Mmset-*targeting shRNA (*n* = 2–3 independent experiments, mean ± s.d., unpaired *t* test: ns, not significant; *p ≤ 0.05). **b** Representative metaphase spreads of TRF2ts MEFs transduced as indicated, collected after 24 h at 39 °C for telomere-FISH. Scale bar, 10 μm. **c** Immunoblot of TRF2ts MEFs or LigIV^−/−^ TRF2ts MEFs used in (**a**), γtubulin serves as loading control. **d** Immunoblot of MMSET and H3K36me2 levels in MEFs used in (**e**). **e** Chromosomal fusions in wild-type (WT) MEFs transduced with control or *Mmset*-targeting shRNA prior to treatment with control or *Trf2*-targeting shRNA for 5 days to induce telomere uncapping. Control transduced *53bp1*^−/−^ and *Rif1*^−/−^ MEFs were included for comparison. Values were normalized for the control condition (*n* = 2 independent experiments, mean ± s.d., unpaired *t* test: **p ≤ 0.01). **f** Telomeric G-overhang quantification (*n* = 2 independent experiments, mean ± s.d., unpaired *t* test: *p ≤ 0.05). **g** Representative telomeric G-overhang assay in control and *Mmset* knockdown TRF2ts MEFs after 48 h at the nonpermissive temperature (37 °C). **h** Chromosome fusions in TRF1^F/F^TRF2^F/F^Ku70^−/−^p53^−/−^ MEFs treated with DMSO or PARPi (Olaparib, 0.5 µM), or transduced with control virus or shRNA targeting *Mmset*. Cells were treated with tamoxifen (4-OHT) for 24 h to induce deletion of floxed shelterin alleles and harvested after a total of 4 days. A minimum of 1500 chromosomes (−4OHT) or 2000 chromosomes (+4OHT) was counted per condition per individual experiment. Genotypes were blinded before manual scoring (*n* = 2 independent experiments, mean ± s.d., unpaired *t* test: ns, not significant; **p ≤ 0.01).

Upon telomere uncapping, the activated DDR triggers NHEJ-mediated ligation of the deprotected chromosome ends at their telomeres [33]. To address whether MMSET functions in NHEJ at uncapped telomeres we inactivated TRF2-mediated telomere protection for 24 h in MMSET-depleted TRF2ts MEFs and analyzed the number of fused chromosomes using telomere fluorescence in-situ hybridization (FISH). Interestingly, MMSET-depleted cells showed a clear reduction in chromosome end fusions, of ~40% (Fig. 2a–c). Also, MMSET-depleted wild-type MEFs transduced with an shRNA targeting *Trf2* showed significantly reduced telomere fusion (Fig. 2d, e, Supplementary Fig. 2A). Telomeres terminate in G-rich 3′ single-stranded DNA (ssDNA) overhangs that are lost during NHEJ-mediated ligation [15, 34]. In line with their reduction in chromosome fusions, MMSET-depleted cells retained telomeric G-overhangs after 48 h of telomere uncapping (Fig. 2f, g). Moreover, aneuploidy caused by missegregation of chromosomes that fused upon telomere uncapping, was partially alleviated in cells with reduced *Mmset*, as is also observed in cells deficient for c-NHEJ due to *Ligase4* or *Rnf8* inhibition (Supplementary Fig. 2B, C).

We next aimed to understand whether MMSET affects only telomere fusions generated through DNA ligase 4-dependent c-NHEJ or also contributes to DNA ligase 3-dependent alt-NHEJ. At unperturbed telomeres, the shelterin complex represses both c-NHEJ and alt-NHEJ. In addition, alt-NHEJ is repressed by Ku70/80 [33]. Upon TRF2 loss, the vast majority of telomere fusions are mediated through Ligase4-dependent c-NHEJ [34]. Indeed, chromosome end-to-end fusion after 24 h of TRF2 inactivation was strongly repressed in Ligase4-deficient TRF2ts MEFs, compared with Ligase4-proficient TRF2ts MEFs (25 to 3%, Fig. 2a). The smaller percentage of fusions left result from Ligase4-independent alt-NHEJ. These were not further decreased by *Mmset* inhibition, suggesting that MMSET does not contribute to Ligase4-independent alt-NHEJ (Fig. 2a, c). To further address this, we used TRF1^F/F^;TRF2^F/F^;Ku70^−/−^;p53^−/−^;Cre-ER^T2^ MEFs in which tamoxifen-induced loss of TRF1 and TRF2 causes processing of deprotected telomeres by Ligase3- and PARP1-dependent alt-NHEJ [33]. Indeed, chromosomal fusions after 4 days tamoxifen treatment were significantly reduced upon PARP1 inhibition with Olaparib (Fig. 2h). Conversely, *Mmset* depletion (Supplementary Fig. 2D, E) did not reduce these alt-NHEJ mediated chromosomal fusions (Fig. 2h). Likewise, shRNA-mediated inhibition of *Ligase3* or *Parp1*, but not *Mmset*, significantly reduced chromosomal fusions in TRF1^F/F^;TRF2^F/F^;Ku70^-/-^;p53^-/-^;Cre-ER^T2^ MEFs treated with tamoxifen for 5 days (Supplementary Fig. 2F, G). Thus, MMSET does not affect alt-NHEJ at deprotected telomeres. Altogether, our results indicate that MMSET facilitates telomeric 3′ overhang degradation and telomere fusion through Ligase4-dependent c-NHEJ, and contributes to telomere dysfunction-induced aneuploidy.

### MMSET promotes H3K36-dimethylation globally and at subtelomeres

**Fig. 3:**
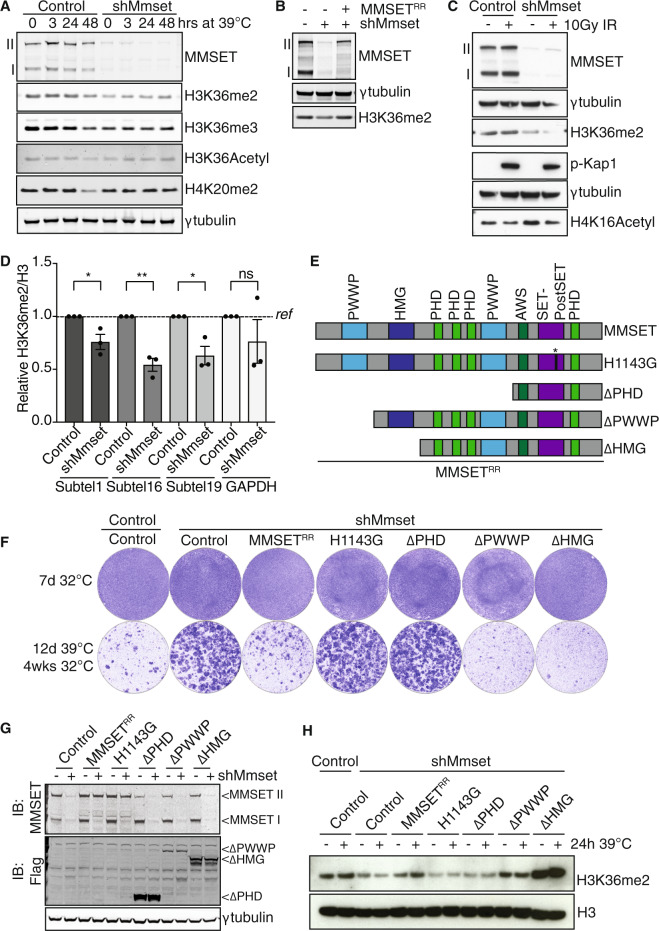
MMSET promotes NHEJ of uncapped telomeres through H3K36 dimethylation. **a** Immunoblot of histone marks in TRF2ts MEFs transduced as indicated and kept at 39 °C for indicated times to induce telomere uncapping. γtubulin serves as loading control. **b** Immunoblot for H3K36me2 in TRF2ts MEFs transduced as indicated and used in Fig. 1e and Supplementary Fig. 1c, in the absence of telomere damage (32 °C). **c** Immunoblot of p53^−/−^ MEFs transduced as indicated, untreated or 20 min post IR (10 Gy). γtubulin serves as loading control. **d** Relative enrichment of H3K36me2/H3 for the indicated target regions in control or shMmset transduced TRF2ts MEFs cultured for 3 h at 39 °C, determined by ChIP for H3 and H3K36me2 and qRT-PCR. QRT-PCRs of individual experiments were performed in technical triplicates and normalized to input DNA. H3K36me2 ChIP data was then normalized for total H3 (*n* = 3 independent ChIP experiments, mean ± SEM, unpaired *t* test: ns, not significant; *p ≤ 0.05; **p ≤ 0.01). **e** Schematic representation of different MMSET cDNA constructs used. H1143G, SET-domain mutant; PHD, plant homeodomain; PWWP, Pro-Trp-Trp-Pro motif; HMG, high mobility-group motif; MMSET^RR^, RNAi-resistant (RR) MMSET. **f** Survival assay of TRF2ts MEFs transduced with indicated shRNA and cDNA constructs and cultured as specified (representative of *n* = 2 independent experiments). **g** Immunoblot showing expression of the different MMSET cDNA constructs. The upper blot, probed with MMSET antibody, shows both full-length MMSET isoform II containing the catalytic domain and the smaller MMSET isoform I that lacks catalytic activity. Both isoforms are targeted by the Mmset shRNA used, while complementation with RNAi-resistant full-length MMSET cDNA (wild-type or H1143G mutant) only restores full-length MMSET isoform II expression. The PHD, PWWP and HMG truncated MMSET variants lose the MMSET antibody epitope and are detected by a Flag-antibody in de lower blot. **h** Immunoblot for H3K36me2 in histone extracts of TRF2ts MEFs transduced as indicated and cultured at 32 °C or for 24 h at 39 °C. H3 serves as loading control (representative of 2 independent experiments).

MMSET is a SET-domain containing HMT. Despite conflicting reports on its catalytic activity, its primary activity appears to be H3K36 mono- and dimethylation [22, 24, 35]. Indeed, *Mmset* depletion caused consistent reduction of global H3K36-dimethylation (H3K36me2) (Fig. 2c, d, Supplementary Fig. 2D), in line with previous reports [24, 30, 36, 37]. This decrease was observed both in absence and presence of telomere uncapping, or DNA damage induced by irradiation (IR), and restored by expressing shRNA-resistant full-length MMSET (Fig. 3a–c, Supplementary Fig. 3A, B). Of note, global levels of H4K20-dimethylation, H3K9-trimethylation or H3K36-acetylation were unaffected (Fig. 3a, Supplementary Fig. 1B). In addition, in both MMSET-depleted MEFs and *Mmset*-knockout MEFs H3K36-monomethylation (H3K36me1) was decreased and, in line with previous studies, also H3K36-trimethylation (H3K36me3, Fig. 3a, Supplementary Fig. 3A, B) [22, 36]. Since H3K36me3 is exclusively catalyzed by SETD2/HYPB [38, 39], we hypothesize that the reduced H3K36me3 upon MMSET loss might be a consequence of less H3K36me2 substrate being available for SETD2-mediated trimethylation.

To understand whether the H3K36me2 decrease in MMSET-depleted cells also occurs at (sub)telomeric regions, we performed chromatin immunoprecipitation (ChIP) experiments with histone H3 and H3K36me2 antibodies, followed by quantitative real-time PCR (qRT-PCR) for subtelomeric regions on chromosome 1, 16, or 19, or a GAPDH control region. Interestingly, in MMSET-depleted cells H3K36me2 was significantly reduced at all three subtelomeric regions (Fig. 3d, Supplementary Fig. 3C). This indicates that the global reduction in H3K36me2 in the absence of MMSET also affects H3K36me2 at (sub)telomeric regions and we hypothesize that by doing so it impacts on DNA repair at deprotected telomeres. Although for reasons of assay sensitivity we cannot exclude a very local or subtle change in H3K36me2 close to the telomere end, we could not detect an increase in H3K36me2 at (sub)telomeric regions upon telomere deprotection (Supplementary Fig. 3D). In line with this, we detected limited association of MMSET with telomeres by ChIP, that was not detectably enhanced upon 3 h of telomere uncapping (Supplementary Fig. 3E, F).

### H3K36-dimethylation by MMSET correlates with NHEJ-efficiency

We next asked whether the H3K36-methyltransferase activity of MMSET, enabled by its SET domain, is important for MMSET activity in promoting telomere fusions. We generated shRNA-resistant mutant versions of MMSET, confirmed their expression and tested their ability to promote genomic crisis in TRF2ts cells depleted for endogenous MMSET (Fig. 3e–g). Exogenous full-length wild-type MMSET abolished the survival of MMSET-depleted TRF2ts MEFs subjected to prolonged telomere uncapping, as seen before in growth curves (Fig. 1e). Interestingly, a catalytically inactive MMSET (SET-domain mutant, H1143 G [22]) and a mutant MMSET lacking its PHD (plant homeodomain) zinc finger domain, important for binding methylated residues [38], were both unable to abolish survival of MMSET-depleted cells. This indicates that the methyltransferase activity as well as the PHD domain are essential for MMSET to promote telomere-NHEJ. On the contrary, the N-terminal PWWP (Pro-Trp-Trp-Pro) chromatin-interacting domain [38] and HMG (high-mobility group) domain are dispensable for MMSET function in promoting genomic crisis upon telomere uncapping, as cells expressing MMSET mutants lacking these domains (ΔPWWP or ΔHMG) efficiently arrested and died upon telomere uncapping. Indeed, the PWWP and HMG domain mutants of MMSET efficiently restored NHEJ-mediated telomere fusion in MMSET-depleted cells, whereas the H1143G and PHD domain mutants did not (Supplementary Fig. 3G).

To further address whether the observed cellular responses to telomere uncapping correlated with H3K36me2 levels, we assessed H3K36me2 levels in histone fractions of MMSET-depleted cells complemented with the various MMSET expression constructs (Fig. 3h). Again, the decrease in H3K36me2 in MMSET-depleted cells was rescued by exogenous wild-type MMSET. Interestingly, both the SET-mutant and PHD-truncated form (ΔPHD) were unable to restore H3K36me2 levels, while expression of the ΔPWWP or ΔHMG MMSET mutants restored or even enhanced H3K36me2. Thus, the ability of the different MMSET mutants to restore H3K36me2 levels directly correlates with their effect on cell survival and chromosomal fusions after telomere uncapping. Although we cannot exclude potential additional activities, this suggests that MMSET facilitates c-NHEJ at deprotected telomeres by promoting H3K36me2 through its SET and PHD domains.

### MMSET is dispensable for early DDR signaling at uncapped telomeres

**Fig. 4:**
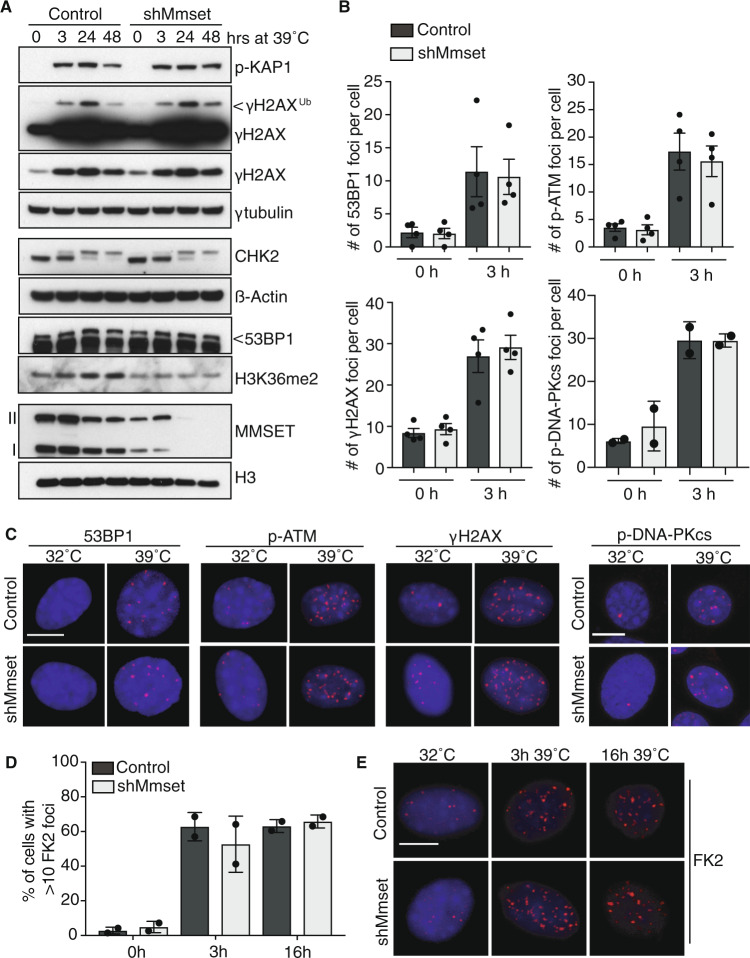
MMSET does not affect DNA damage signaling at unprotected telomeres. **a** Immunoblots of different DDR-factors in TRF2ts MEFs, control infected or transduced with an shRNA targeting *Mmset* and subjected to telomere uncapping at 39 °C for the indicated times. γtubulin, ß-actin, and H3 serve as loading controls (representative blots of a minimum of two independent experiments). **b** Quantification of 53BP1 foci, p-ATM foci, γH2AX foci, and p-DNA-PKcs foci in TRF2ts MEFs in undamaged conditions (32 °C; 0 h) or after 3 h of telomere deprotection (39 °C; 3 h). (*n* = 4 independent experiments, mean ± SEM, a minimum of 312 (53BP1), 316 (p-ATM), or 158 (γH2AX) cells were quantified in each condition and per experiment; *n* = 2 for p-DNA-PKcs, mean ± s.d., a minimum of 81 cells per condition per experiment). **c** Representative images of foci quantified in (**b**). Scale bars, 10 μm. **d** Quantification of FK2 foci in TRF2ts MEFs transduced with an shRNA targeting *Mmset* or control, and placed at the nonpermissive temperature of 39 °C for 3 or 16 h to induce telomere uncapping. In each experiment a minimum of 178 cells were quantified per condition (*n* = 2 independent experiments, mean ± s.d.). **e** Representative images of cells quantified in (**d**). Scale bar, 10 μm.

As both MMSET and H3K36-methylation have been associated with transcriptional control [22], we first considered that MMSET might indirectly affect telomere-NHEJ through transcriptional regulation of DDR-factors. However, MMSET depletion did not significantly affect mRNA levels of various DDR-factors tested, including factors known to be important for telomere-NHEJ, like RNF8, RNF168, 53BP1, MAD2L2, LIG4 (DNA ligase 4), and XRCC4 (Supplementary Fig. 4A, B). In addition, MMSET depletion did not affect RAD51, 53BP1 and LIG4 protein levels (Fig. 4a, Supplementary Fig. 4C, D). Although we cannot exclude minor effects or effects through transcriptional regulation of genes not addressed, these results supported examination of other potential ways by which MMSET might affect NHEJ at telomeres. We therefore addressed the activation of DNA damage signaling responses. While MMSET-depleted TRF2ts MEFs showed decreased H3K36me2 prior to and during telomere uncapping, phosphorylation of KAP1, H2AX, or CHK2 at 3 or 24 h of telomere deprotection was unaffected by MMSET depletion (Fig. 4a). We interpret the slightly higher KAP1 and H2AX phosphorylation in MMSET-depleted cells at 48 h of telomere uncapping as a consequence of reduced repair by NHEJ, while in control cells DNA damage signaling declines upon telomere-NHEJ. Also IR-induced KAP1, H2AX, and CHK2 phosphorylation were similar in control and MMSET-depleted cells (Supplementary Fig. 4E). We also analyzed subnuclear foci of H2AX phospho-S139 (γH2AX), ATM phospho-S1981 (p-ATM), and conjugated ubiquitin that spreads over chromatin surrounding sites of DNA damage and is detectable with FK2 antibody [40]. Telomere deprotection for 3 h led to clear accumulation of γH2AX, p-ATM, and FK2 signal into foci, that were not different in number between MMSET-depleted cells and control cells (Fig. 4b–e, Supplementary Fig. 4F). Together these results indicate that MMSET does not impact on the recognition of uncapped telomeres and early signaling responses by the DDR-machinery.

Downstream of these early DDR responses is the control of DNA repair pathway choice by 53BP1 and BRCA1, in which 53BP1 inhibits DNA end-resection to facilitate NHEJ while BRCA1 promotes end-resection to generate 3′ ssDNA-overhangs and direct DNA repair by HR [32, 33, 41–47]. MMSET has been implicated in the recruitment of 53BP1 to DNA damage via induction of H4K20me2 [28, 29]. However, additional reports showed that MMSET does not have specific activity toward H4K20me2 and does not affect 53BP1 recruitment [5, 48, 49]. In line with the latter reports, we did not observe defects in 53BP1 recruitment and accumulation at deprotected telomeres in MMSET-depleted cells by immunofluorescence (IF), or IF-FISH (Fig. 4b, c, Supplementary Fig. 4F, G). Furthermore, global H4K20me2 levels appeared unaffected by MMSET depletion (Fig. 3a). We next investigated if MMSET affects ssDNA formation. Phosphorylation of CHK1 was slightly increased in MMSET-depleted cells, which may indicate increased ATR activation (Supplementary Fig. 5A). However, we did not observe increased phosphorylation of the ssDNA-binding protein RPA upon IR or telomere deprotection (Supplementary Fig. 5A, B), as would be seen upon loss of the end-resection inhibitors 53BP1, RIF1 or MAD2L2 [32, 42, 44]. Also, recruitment of BRCA1 to uncapped telomeres was not significantly altered (Supplementary Fig. 5C, D). Together this suggests that MMSET depletion does not impair NHEJ by enhancing end-resection and ssDNA formation.

As upstream DDR responses are unperturbed in MMSET-depleted cells, we consider it most likely that MMSET affects engagement or activity of the NHEJ-machinery itself. We therefore analyzed the effect of MMSET depletion on DNA-PKcs autophosphorylated on S2056 (p-DNA-PKcs), important for the ligation of DNA ends during NHEJ [50, 51]. Telomere deprotection for 3 h led to clear accumulation of p-DNA-PKcs subnuclear foci that were not different in number between MMSET-depleted cells and control cells, indicating that NHEJ is not affected by MMSET at the level of DNA-PKcs localization and autophosphorylation (Fig. 4b, c, Supplementary Fig. 4F). Other NHEJ-components do not accumulate robustly enough at telomeres to be detected as clearly discernable foci by IF. We therefore aimed to further address telomeric localization of NHEJ-components by ChIP. As MMSET was previously connected to the recruitment of XRCC4 to DNA damage [30], we attempted to assess telomeric-localization of XRCC4 by ChIP, using either qRT-PCR with subtelomere-specific primers or dot blot detection of TTAGGG-telomere repeats. However, the signals retrieved for XRCC4-ChIP at (sub)telomeres were too low and not detectably increased upon telomere deprotection, precluding proper assessment of the potential effect of MMSET on XRCC4 localization to uncapped telomeres. This probably relates at least in part to the long 30–100 kb telomere-repeat stretches at mouse telomeres that complicate assessment of NHEJ-factors acting at telomere ends and not spreading extensively over telomeres, by ChIP. Therefore, although our data are most compatible with MMSET-dependent H3K36me2 being important for downstream steps in NHEJ at telomeres, such as those immediately preceding or at the actual DNA ligation step by the XRCC4/Lig4-complex, the precise mechanism remains unclear at this point.

### Additional H3K36me2-specific HMTs contribute to NHEJ at uncapped telomeres

**Fig. 5:**
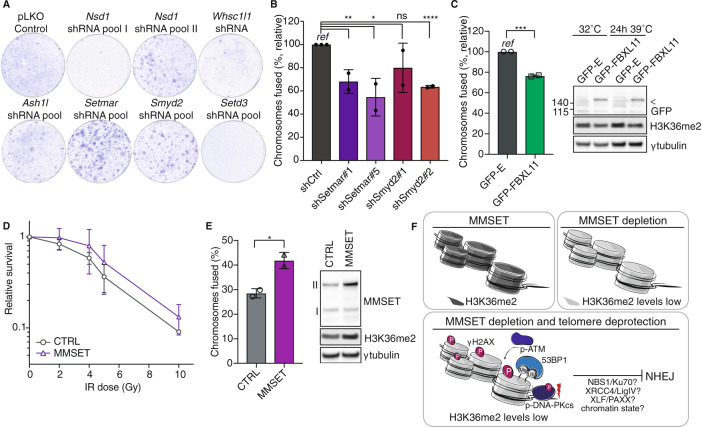
Additional H3K36me2-specific HMTs contribute to NHEJ at uncapped telomeres. **a** Survival assay of TRF2ts cells transduced with the indicated lentiviral shRNA-pools or pLKO control vector. After 12 days at 39 °C cells were returned to 32 °C for 4 weeks and stained with crystal violet. **b** Chromosome fusions in TRF2ts MEFs transduced with indicated lentiviral shRNAs (‘shCtrl’: scrambled or luciferase control shRNA). A minimum of 1470 chromosomes was counted per condition per experiment and counts were normalized to shCtrl (*ref*) (*n* = 2 independent experiments (except shCtrl, *n* = 3), mean ± s.d., unpaired *t* test: ns, not significant; *p ≤ 0.05; **p ≤ 0.01; ****p ≤ 0.0001). **c** Left: chromosomal fusions upon 24 h of telomere uncapping at 39 °C in TRF2ts MEFs transduced with GFP-tagged FBXL11 or GFP-expressing control vector (“GFP-E”). A minimum of 1800 chromosomes was counted per condition per experiment (*n* = 2 independent experiments, mean ± s.d., unpaired *t* test: ***p ≤ 0.001). Right: corresponding immunoblot for GFP-FBXL11 (arrow) and H3K36me2, γtubulin serves as loading control. **d** Relative survival of TRF2ts MEFs transduced with a MMSET expression (“MMSET”) or empty (“CTRL”) viral vector. Cells were irradiated with the indicated doses (Gy) and left to recover for 10 days prior to crystal violet staining and quantification (*n* = 2, mean ± s.d., cells were plated as technical duplicate). **e** Left: chromosomal fusions in TRF2ts MEFs transduced as in (**d**). A minimum of 2000 chromosomes was counted per condition per experiment (*n* = 2 independent experiments, mean ± s.d., unpaired *t* test: *p ≤ 0.05). Right: corresponding immunoblot for MMSET and H3K36me2, γtubulin serves as loading control. **f** Model on how MMSET could affect NHEJ at telomeres through H3K36me2.

In mammalian cells, multiple SET-domain containing enzymes can catalyze H3K36me1 and H3K36me2 [38]. To address whether additional H3K36me2-specific HMTs affect NHEJ at telomeres we made shRNA-pools against the following HMTs: [38] *Nsd1* (9 shRNAs in 2 pools), *Ash1l*, *Setmar*, *Smyd2*, *Setd3* (for each 4-5 shRNAs/pool), and *Whsc1l1* (1 shRNA). These shRNA-pools were tested for enabling TRF2ts MEFs to avoid telomere NHEJ-related genomic crisis. Interestingly, inhibition of *Setmar* or *Smyd2* with shRNA-pools gave TRF2ts cells a survival benefit under telomere uncapping conditions (Fig. 5a). Moreover, depleting *Setmar* or *Smyd2* using individual shRNAs decreased fusion of uncapped telomeres (Fig. 5b), indicating that also SETMAR and SMYD2 act in facilitating telomere-NHEJ. In fact, SETMAR has been implicated before in NHEJ in a different setting, indicating a more general role in NHEJ [52]. The identification of additional H3K36me2-specific HMTs that contribute to NHEJ at uncapped telomeres further emphasizes the importance of this chromatin modification in DNA repair and suggests that a certain degree of redundancy is at play.

Several lysine demethylases act in removing H3K36me2. We hypothesized that overexpressing such a H3K36me2-specific lysine demethylase would mimic MMSET depletion. Indeed, overexpression of the H3K36me2-specific demethylase FBXL11 (a.k.a KDM2A or JHDM1A [53]) decreased fusion of uncapped telomeres in TRF2ts MEFs, further confirming the importance of H3K36me2 in telomere-NHEJ (Fig. 5c).

Finally, while compromised NHEJ due to lack of MMSET might sensitize cells to different types of DNA damage [28, 29], we hypothesized that cells expressing higher MMSET levels might have enhanced NHEJ capabilities, allowing them to cope better with IR-induced DNA damage. Indeed, MMSET overexpression made cells more resistant to IR (Fig. 5d), in line with recent work showing that MMSET-high (t4;14)+ myeloma cells proliferate better upon treatment with DNA-damaging drugs than cells expressing wild-type MMSET levels [30]. Moreover, overexpression of MMSET in TRF2ts MEFs raised H3K36me2 levels and increased telomere-NHEJ (Fig. 5e), further indicating that elevated MMSET levels enhance NHEJ-mediated DNA repair.

## Discussion

Here, we describe an important role for MMSET in facilitating c-NHEJ at deprotected telomeres. Our data suggest a role for MMSET-dependent H3K36me2 at late stages of the NHEJ process, downstream of 53BP1-controlled DNA repair pathway choice and independent of DNA-PKcs localization and autophosphorylation (Fig. 5f). MMSET has been implicated in DNA repair before. MMSET-depleted cells display impaired DNA repair in different cellular assays and increased sensitivity toward multiple DNA-damaging agents [28–30, 54, 55]. Although this points at a global role of MMSET in DNA repair, to what extent these phenotypes can be attributed specifically to NHEJ is not well established and also the underlying mechanism has been under debate. MMSET has been implicated in immunoglobulin class-switch recombination (CSR), a physiological process relying on c-NHEJ-mediated joining of induced DSBs [54, 55]. However, this seems largely attributable to MMSET affecting the generation of DSBs during CSR, rather than their end-joining. Furthermore, MMSET was reported to facilitate 53BP1 recruitment through de novo H4K20-dimethylation, thereby promoting 53BP1-directed repair [28, 29]. However, additional work indicated that the primary enzymatic activity of MMSET is toward H3K36, rather than H4K20, and that MMSET is dispensable for 53BP1 recruitment to DNA damage [5, 22, 24, 35, 36, 48, 49]. Our study is in line with the latter reports, as 53BP1 accumulation to uncapped telomeres was not affected by MMSET depletion, nor was end-resection detectably elevated, indicating that 53BP1’s activity in inhibiting end-resection is preserved. Also, complementation assays with MMSET mutants showed that regulation of telomere-NHEJ by MMSET correlates directly with its effect on H3K36me2. Via H3K63me2, MMSET might promote telomere-NHEJ in multiple potential ways.

First, MMSET might indirectly facilitate NHEJ, in which changes in global H3K36me2 levels or the presence of MMSET itself affect transcriptional regulation of DDR-proteins. Indeed, MMSET isoforms were shown to function in transcriptional regulation and interact with HDAC1 and HDAC2 transcriptional corepressors [22, 56–58]. Furthermore, MMSET overexpression in t(4;14)+ multiple myeloma cells changes the genomic distribution of H3K36me2 and affects gene expression [24, 36, 59]. While our data do not exclude that MMSET affects NHEJ through transcriptional regulation, we did not observe significant impact of MMSET depletion on the levels of various DDR-proteins critical in NHEJ or on activation and recruitment of p-ATM, γH2AX, 53BP1, BRCA1, and p-DNA-PKcs (Fig. 4). Thus, if there would be any effect by MMSET on NHEJ through transcriptional changes, this would most likely be relevant to steps further downstream or potential alternative mechanisms in DNA repair control.

Second, H3K36me2 could function as platform for DNA repair protein recruitment. H3K36 methylation has been linked to different DNA repair pathways. While SETD2-dependent H3K36me3 pre-exists and is highly enriched at actively transcribed genes where it channels DNA repair through HR [39, 60–62], H3K36me2 has been associated with NHEJ [52, 63–65]. H3K36me2 induced around DSBs by SETMAR (a.k.a. Metnase) promotes association and stabilization of Ku70 and NBS1 at DSBs, thereby enhancing repair by NHEJ [52]. NBS1 interacts with H3K36me2 in vitro and could potentially respond directly to increased H3K36me2 at DSBs [65]. The demethylase FBXL11 (KDM2A/JHDM1A) counteracts H3K36me2 and is inactivated upon DNA damage to allow H3K36me2 and efficient repair [52, 65]. Our work indicates that a SETMAR-controlled mechanism is also at play at deprotected telomeres, but unclear is if H3K36me2 is induced at deprotected telomeres by SETMAR or other H3K36me2-HMTs. Our ChIP experiments indicated that H3K36me2 pre-exists at telomeres and did not detect increased H3K36me2 at (sub)telomeres upon telomere deprotection. However, we cannot exclude a local increase in H3K36me2 at the distal ends of deprotected telomeres that remains below detection in our ChIP experiments covering long telomeric distances. A limited local increase could underlie the increased H3K36me2 occasionally visible in immunoblots of TRF2ts cells undergoing telomere uncapping (e.g., Figure 4a). Whether H3K36me2-facilitated telomere-NHEJ involves effects on telomeric localization of Ku is difficult to discern since, besides binding DNA ends, Ku also localizes to telomeres via association with TRF1 and TRF2 [66–69]. Furthermore, it seems unlikely that NBS1 at telomeres is considerably affected by MMSET as MRN-dependent ATM activity was unperturbed in MMSET-depleted cells. While our data indicate that MMSET-dependent H3K36me2 is most likely important for further downstream steps of NHEJ at telomeres, such as those immediately preceding or at the actual DNA ligation step by XRCC4/Lig4, DNA-PKcs localization and autophosphorylation appeared normal in MMSET-depleted cells. Nevertheless, other components of the NHEJ-machinery could potentially be affected by MMSET and H3K36me2, e.g., XLF, PAXX, or XRCC4/Lig4. As MMSET was previously shown to facilitate XRCC4 recruitment to DNA damage [30], XRCC4 is a plausible candidate for being controlled by MMSET activity at deprotected telomeres. Unfortunately, technical limitations prohibited us from concluding whether MMSET promotes XRCC4 recruitment also to deprotected telomeres. As similar limitations apply to other NHEJ-components, the precise NHEJ-components at telomeres affected by MMSET remain elusive for now.

Third, MMSET and its role in H3K36 methylation could impact on DNA repair by affecting global chromatin state. Both loss of yeast Set2 and depletion of mammalian MMSET were reported to alter chromatin accessibility [36, 63, 64]. Furthermore, the histone deacetylases HDAC1 and HDAC2, which interact with MMSET, have also been implicated in NHEJ, including at uncapped telomeres [16, 70]. Whether MMSET via interactions with HDACs affects acetylation at telomeres or whether an interplay between methylation and acetylation influences chromatin states at telomeres and thereby DNA repair, would be interesting to further investigate.

Finally, while our data indicate that MMSET impacts on telomere-NHEJ primarily through H3K36-methylation, it is possible that also nonhistone targets of MMSET contribute to c-NHEJ. Interestingly, MMSET was recently shown to dimethylate PTEN upon DNA damage, thereby aid PTEN recruitment to DNA damage via interaction with 53BP1, support PTEN phosphatase activity toward H2AX and affect sensitivity to DNA-damaging agents [71]. Although H2AX phosphorylation was not clearly affected in MMSET-depleted cells undergoing telomere uncapping, to what extent MMSET-mediated methylation of PTEN or other potential nonhistone targets contribute to telomere-NHEJ is interesting to explore in future research.

Interestingly, our results indicate that while critical for c-NHEJ, MMSET is dispensable for alt-NHEJ. The contribution of chromatin marks to c-NHEJ versus alt-NHEJ is still unclear. As alt-NHEJ shares the initial DNA resection steps with HR, it is conceivable that a chromatin environment facilitating resection and HR could also aid in alt-NHEJ [72]. However, while SETD2-dependent H3K36me3 promotes HR at active genes, SETD2 loss increased alt-NHEJ rather than impaired it, with alt-NHEJ serving as backup for ineffective HR [60, 62]. In our work, MMSET loss caused decreased H3K36me3, but did not increase alt-NHEJ at deprotected telomeres. Although high alt-NHEJ rates in our experimental system might technically prohibit detecting a further increase, we hypothesize that the effect of SETD2-mediated H3K36me3 on alt-NHEJ might be restricted to defined chromatin locations, such as active genes, and not apply to telomeres.

Aside from multiple myeloma, MMSET is overexpressed in a variety of cancers [73]. By promoting NHEJ, MMSET overexpression could potentially contribute to tumorigenesis through telomere-induced genomic instability or inappropriately favoring NHEJ for DNA repair, thereby elevating the risk of genomic translocations or other alterations. Indeed, MMSET overexpression increased NHEJ-efficiency at deprotected telomeres (Fig. 5e). Moreover, MMSET overexpression could potentially increase overall DNA repair-efficiency, which in established tumors might affect the efficacy of DNA-damaging therapies. Indeed, MMSET overexpressing cells show resistance to chemotherapy [30] and decreased sensitivity to IR (Fig. 5d). Therefore, inhibition of MMSET by small-molecule inhibitors in combination with DNA-damaging chemotherapeutics or IR could potentially be potent in treatment of cancers with increased MMSET expression. Better understanding of the mechanism by which MMSET functions in DNA repair will help further improve treatment opportunities for cancers with altered MMSET expression.

## Material and methods

### Cells, growth and survival assays

*Trf2*^*-/-*^*;p53*^*-/-*^*;*TRF2ts MEFs [18] were maintained at 32 °C, Phoenix-eco cells, 293 T (ATCC), and other MEFs at 37 °C.

For short-term growth assays, TRF2ts MEFs were plated in triplicate at 5000 cells/well on 12-well plates and allowed to adhere overnight at 32 °C. Cells were incubated at 39 °C to induce telomere uncapping for 12 days, fixed every 3 days using 4% formaldehyde and stained with 0.1% crystal violet. For quantification crystal violet was extracted with 10% acetic acid and absorbance at 595 nm was measured in a Tecan microplate reader.

For survival assays TRF2ts MEFs were plated at 45,000 cells/10 cm plate or 150,000 cells/15 cm plate, incubated at 39 °C for 12 days and returned at 32 °C for 2–4 weeks until crystal violet staining. One set of plates was stained after 7 days at 32 °C to control for potential toxicity of shRNAs. Culture details, viral transduction, cell cycle, and aneuploidy analysis by flow cytometry are detailed in Supplementary Materials and Methods.

### Methyltransferase screen

*Trf2*^*-/-*^*;p53*^*-/-*^*;*TRF2ts MEFs were infected with a custom-made pRetrosuper-shRNA library targeting 38 known or predicted methyltransferases with 4 shRNAs per gene, or with empty pRetrosuper. Library DNA was divided into 7 pools, viruses were produced per pool and infected separately. ShRNA sequences for the methyltransferase library and H3K36me2-HMTs are in Supplementary Materials and Methods. After selection for puromycin-resistance cells were plated (150,000/15 cm dish) for survival assays.

### Telomere-fusion and G-overhang analysis

Telomere-fusion assays using telomere-FISH were performed essentially as before [18, 32] and are further detailed in Supplementary Materials and Methods.

3′ single-stranded G-overhangs were analyzed using pulsed-field gel electrophoresis and in-gel hybridization with ^32^P-labeled (CCCTAA)_4_-oligonucleotide as before [18, 32].

### qRT-PCR

Total RNA extraction using TRIzol (Ambion), reverse transcription and qRT-PCR were performed using standard procedures and described in Supplementary Materials and Methods.

### Immunoblotting and histone extraction

Preparation of whole-cell lysates and immunoblotting were done as before [32]. Primary and secondary antibodies and immunodetection methods are listed in Supplementary Materials and Methods.

For acid extraction of core histones, cells pellets were harvested and sequentially extracted with perchloric acid and HCl, followed by precipitation of core histones with trichloroacetic acid. Histone pellets were sequentially washed with 100% Aceton/0.006% HCl and 100% Aceton, dried, and resuspended for protein concentration measurement (Bradford assay) and loading onto precast SDS-PAGE gels for immunoblotting. Additional details are in Supplementary Materials and Methods.

### IF, IF-FISH

TRF2ts MEFs were seeded onto eight-well chamber slides (Millipore). For IF detection of γH2AX, p-ATM, and 53BP1, cells were washed with PBS and fixed for 10 min with 2% paraformaldehyde (PFA). For p-DNA-PKcs, FK2 and BRCA1, cells were washed with PBS and pre-extracted with ice-cold 0.5% Triton/PBS on ice for 5 min prior to fixation in 2% PFA. Subsequent processing was done as before [32], further specified in Supplementary Materials and Methods.

For IF-FISH cells were pre-extracted with 0,5% triton/PBS, fixed for 10 min with 2% PFA and 10 min on ice with methanol. Staining with primary antibody for 53BP1 (NB100-305, Novus, 1:500) and secondary antibody (Alexa Fluor 568, Invitrogen), FISH detection of telomere repeats with a FITC-OO-(CCCTAA)_3_ PNA custom probe (Biosynthesis) and image acquisition were done as described [18].

### Chromatin immunoprecipitation

*Trf2*^*-/-*^*;p53*^*-/-*^*;*TRF2ts MEFs were crosslinked for 15 min with 1% formaldehyde. After 5 min in 0.2 M Glycine and three washes with ice-cold PBS, cells were resuspended in 2 ml lysis buffer with iodoacetamide, sodium butyrate, protease and phosphatase inhibitors. For chromatin preparation, 1 ml triton dilution buffer (with inhibitors above) was added and lysates were sonicated on ice. ChIP was performed overnight at 4 °C with IgG rabbit isotype control or H3K36me2, H3, or GFP antibodies pre-coupled to protein A and protein G magnetic beads. After multiple washes immunoprecipitated protein-DNA complexes were eluted, crosslinks were reversed and DNA was recovered and analyzed for (sub)telomeric DNA content by qRT-PCR or dot blots.

Further details are in Supplementary Materials and Methods.

### Statistics

Statistical analyses were performed with GraphPad Prism using an unpaired two-tailed Student’s *t* test. Significances: not significant (ns) *p* > 0.05; *p ≤ 0.05; **p ≤ 0.01; ***p ≤ 0.001; ****p ≤ 0.0001.

## Supplementary information

Supplementary Figures

Supplementary Materials and Methods
