# Supplementary material for: H3K36 dimethylation by MMSET promotes classical non-homologous end-joining at unprotected telomeres
Source: Oncogene. 2020 May 29;39(25):4814–27. doi: 10.1038/s41388-020-1334-0 (PMC7299843; doi:10.1038/s41388-020-1334-0)
Supplement: Supplementary file 1 — Supplementary Figures [file 41388_2020_1334_MOESM1_ESM.pdf]

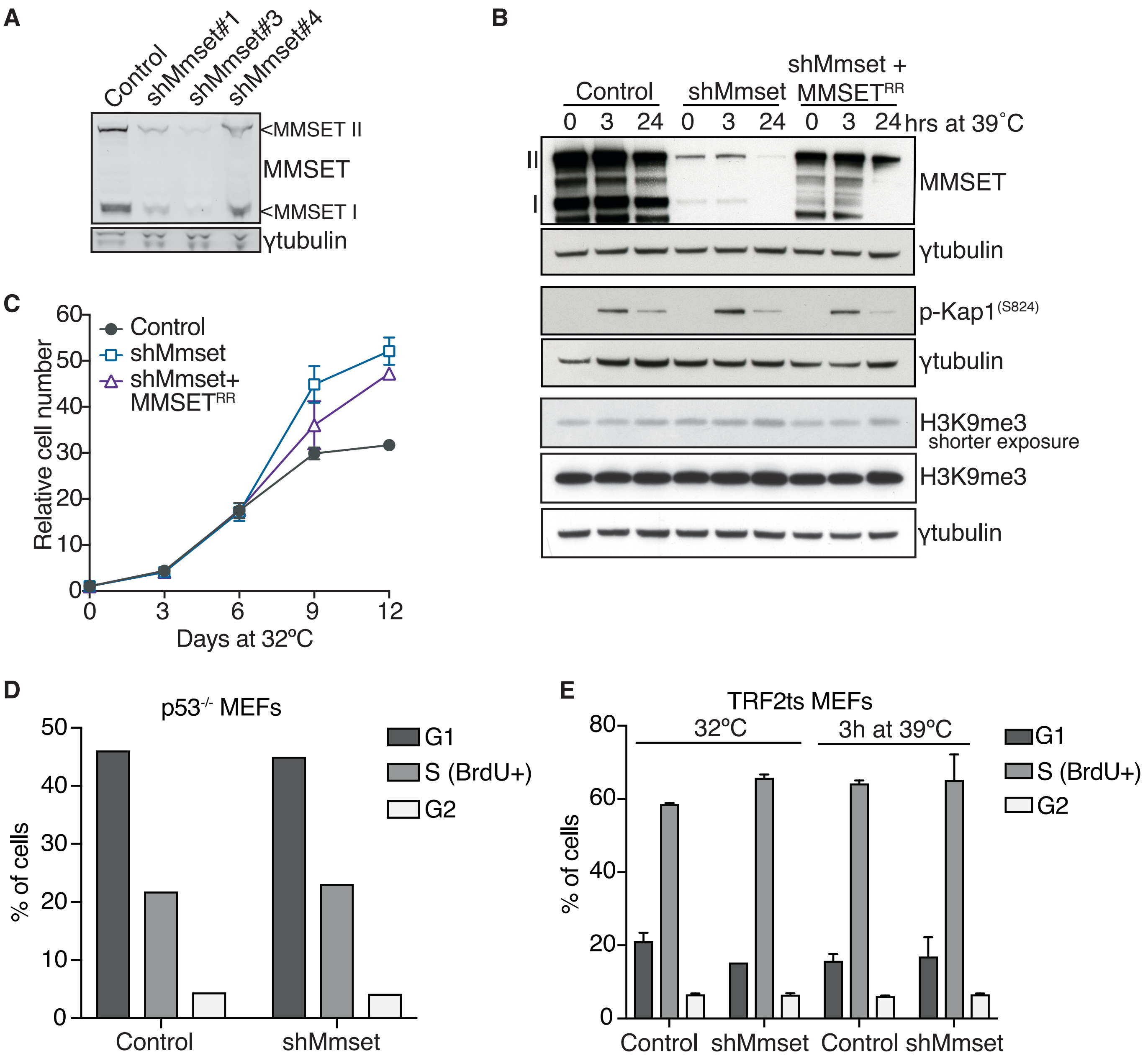

Supplementary figure 1. MMSET identified as a novel telomere-induced genomic instability regulator. A. Immunoblot for MMSET in TRF2ts MEFs used in the survival assay in Fig. 1D. The upper band indicates the full-length MMSET isoform II, containing catalytic activity. The lower band represents the shorter MMSET isoform I, lacking catalytic activity. **B.** Immunoblot of TRF2ts MEFs cultured at 32°C or for the indicated times at 39°C to induce telomere uncapping. γtubulin serves as loading control. **C.** Growth curves of *Mmset* knockdown and complemented cells used in Fig. 1E, at the permissive temperature of 32°C (data represent mean±s.d. of a technical triplicate). **D.** Cell cycle analysis by flow cytometry of $p53^{-/-}$ MEFs transduced with control or *Mmset*-targeting shRNA. BrdU incorporation was used to quantify S-phase cells. **E.** Cell cycle distribution, determined by flow cytometry, of TRF2ts MEFs transduced with control or *Mmset*-targeting shRNA, kept at 32°C or for 3 h at 39°C (n=2, mean±s.d.).

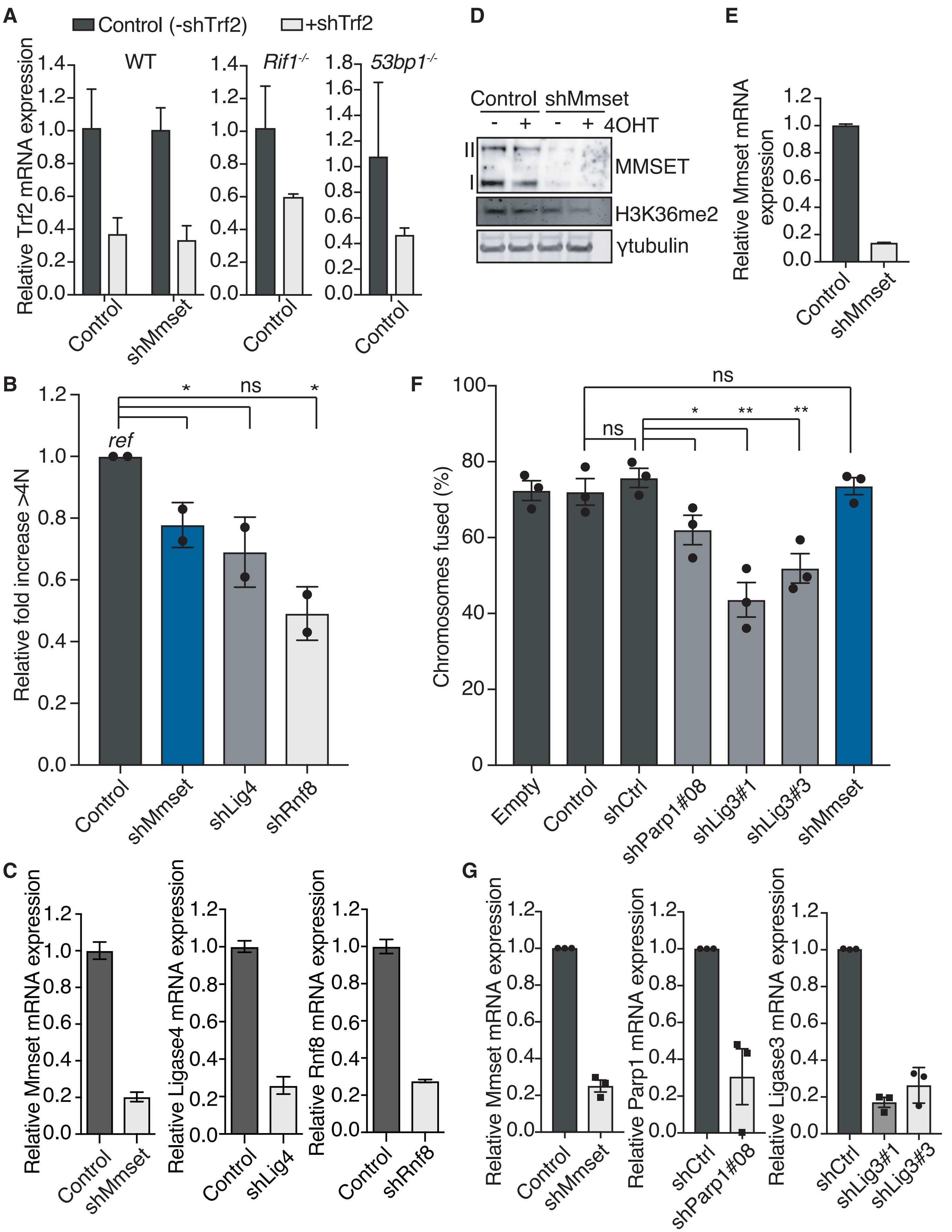

Supplementary figure 2. A. qRT-PCR analysis of *Trf2* mRNA levels in cells used in Fig. 2E. **B.** TRF2ts MEFs transduced with indicated shRNAs were grown at the non-permissive temperature of 39°C for 48 h followed by PI staining and flow cytometry analysis for the cell population with >4N DNA content (n=2 independent experiments, mean±s.d., unpaired t-test: ns, not significant; *p≤0.05; **p≤0.01).

(Legend continues on next page.)

C. Representative qRT-PCR analysis of *Mmset*, *Ligase4* and *Rnf8* mRNA expression levels of cells used in **B**. **D.** Immunoblot of TRF1$^{F/F}$;TRF2$^{F/F}$;Ku70$^{-/-}$;p53$^{-/-}$ MEFs used in Fig. 2H, transduced with indicated shRNA and harvested 4 days after treatment with 4-OHT or left untreated. **E.** Representative qRT-PCR analysis of *Mmset* expression level of cells used in Fig. 2H. **F.** Chromosomal fusions in TRF1$^{F/F}$;TRF2$^{F/F}$;Ku70$^{-/-}$;p53$^{-/-}$ MEFs untransduced ('Empty'), transduced with control ('Control') or *Mmset*-targeting retroviral shRNA, Ligase3- or Parp1-targeting lentiviral shRNAs, or scrambled control lentiviral shRNA ('shCtrl')). Cells were treated with 4-OHT for 5 days prior to metaphase harvest. A minimum of 1700 chromosomes was counted per condition per experiment, genotypes were blinded before scoring (n=3 independent experiments, mean±SEM, unpaired t-test: ns, not significant; p*≤0.05; **p≤0.01). **G.** qRT-PCR analysis of *Mmset*, *Parp1* and *Ligase3* expression levels of cells used in **F** (n=3 independent experiments with technical triplicates each, mean±SEM).

Supplementary figure 3

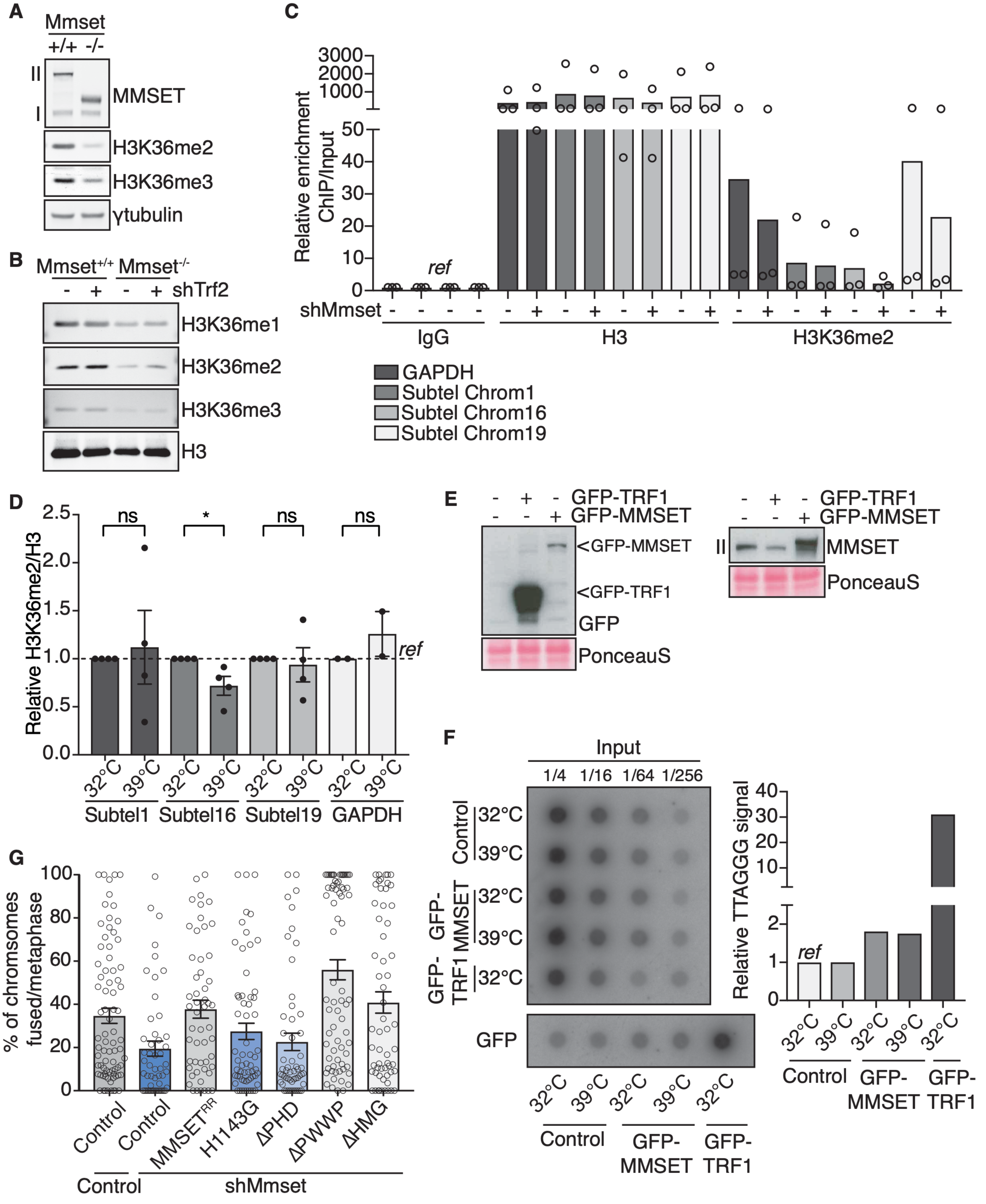

Supplementary figure 3. A. Immunoblot for MMSET, γ-tubulin (loading control), H3K36-dimethylation (me2) and H3K36-trimethylation (me3) levels in WT (+/+) or *Mmset*-knockout MEFs (-/-). **B.** Immunoblot for H3K36me1, H3K36me2 and H3K36me3 levels on histone extracts from MEFs transduced with control or a *Trf2*-targeting shRNA. H3 serves as loading control. **C.** Enrichment of ChIP/Input of data shown in Fig. 3D before correction for total H3 levels and plotted relative to non-specific IgG (=1) control pulldowns (n=3 independent ChIP experiments, with for each the mean shown of technical triplicates normalized to input DNA).

(Legend continues on next page.)

D. Relative enrichment of H3K36me2/H3 for the indicated target regions in TRF2ts MEFs kept at the permissive temperature (32°C) or for 3 h at 39°C to induce telomere deprotection, as determined by ChIP and quantified by qRT-PCR. Dots represent individual ChIP experiments. QRT-PCRs of individual experiments were performed in technical triplicates and normalized to input DNA. H3K36me2 ChIP data was then normalized for total H3. Graph represents the enrichment of H3K36me2/H3 at 39°C, relative to the corresponding 32°C control as indicated (*ref*) (n=4 independent ChIP experiments, except for a GAPDH control region (n=2), mean±SEM, unpaired t-test: ns, not significant; *$p \leq 0.05$). **E.** Immunoblots of TRF2ts MEFs used in **F**, showing expression of GFP-tagged constructs (left) and MMSET (right). PonceauS serves as loading control. **F.** Dot blot for detecting telomeric DNA of input samples (upper blot) or after GFP-ChIP (bottom blot) in TRF2ts MEFs transduced as indicated and kept at 32°C or for 3 h at the non-permissive temperature of 39°C to induce telomere deprotection. Graph (right) represents the quantification of the GFP-ChIP signal corrected for background and input and plotted relative to the 32°C sample in control condition (*ref*). Representative experiment out of three is shown. GFP-TRF1 serves as positive control. **G.** Chromosomal fusions in TRF2ts cells transduced with indicated shRNA and cDNA-constructs. Individual data points represent the percentages of fused chromosomes per metaphase counted. A minimum of 51 metaphases and 2500 chromosomes was counted for each condition. Bars represent the mean±SEM over all data points.

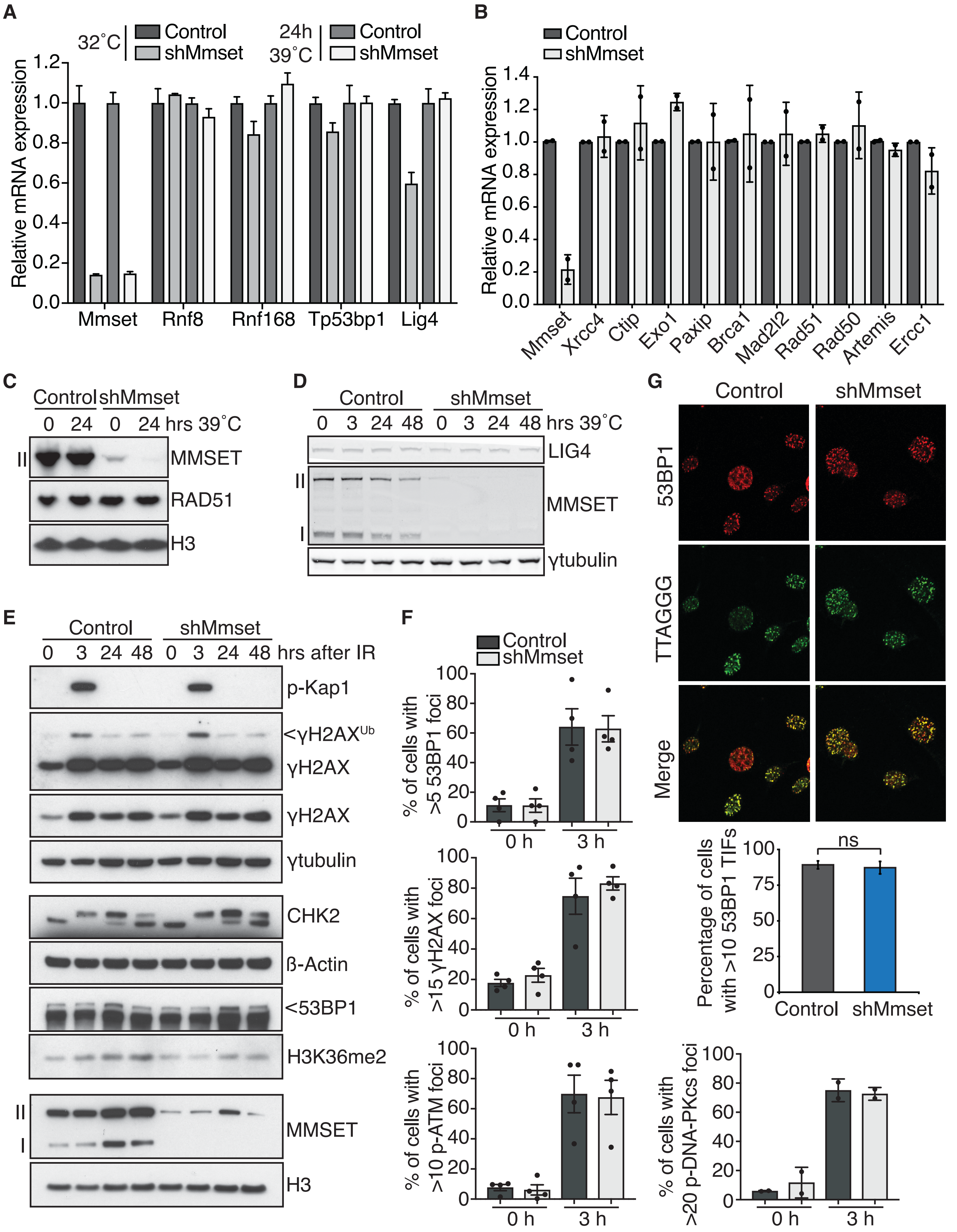

Supplementary figure 4. A. qRT-PCR analysis of mouse *Mmset*, *Rnf8*, *Rnf168*, *Tp53bp1* and *Lig4* mRNA levels in MMSET-depleted or control TRF2ts MEFs. Cells were kept at 32°C or placed at 39°C for 24 h to induce telomere uncapping (technical triplicate, mean±s.d.). **B.** qRT-PCR analysis of mouse mRNA expression levels of various DDR-components in control or MMSET-depleted TRF2ts MEFs (32°C) (n=2 independent experiments with technical triplicates, mean±s.d.). *(Legend continues on next page.)*

C. Immunoblot for MMSET and RAD51 in TRF2ts cells kept at 39°C for 24 h to induce telomere uncapping or at 32°C control conditions. Cells were transduced with indicated shRNAs. H3 serves as a loading control (representative of n=2 independent experiments). **D.** Immunoblot for LIG4, MMSET and γtubulin (loading control) in control or MMSET-depleted TRF2ts MEFs shifted to the non-permissive temperature as indicated. **E.** Immunoblot for various DDR-proteins in control or MMSET-depleted TRF2ts MEFs. Cells were irradiated with 5Gy and harvested at indicated timepoints after irradiation or left unirradiated (0 hrs). H3, γ-tubulin and ß-actin are loading controls. **F.** Different representation of the data shown in Fig. 4C; quantification of DDR foci detected by IF in Control or MMSET-depleted cells (n=4 independent experiments, mean±SEM or n=2 independent experiments for p-DNA-PKcs, mean±s.d.). **G.** Representative IF images of control or shMmset transduced TRF2ts MEFs subjected to 3 h of telomere uncapping at 39°C, stained for 53BP1 by IF and telomere repeats (TTAGGG) by FISH. Merge shows the overlay. Graph shows quantification of 53BP1 foci co-localizing with TTAGGG FISH signals into telomere dysfunction-induced foci (TIFs) (n=5 independent experiments, unpaired t-test: ns, not significant).

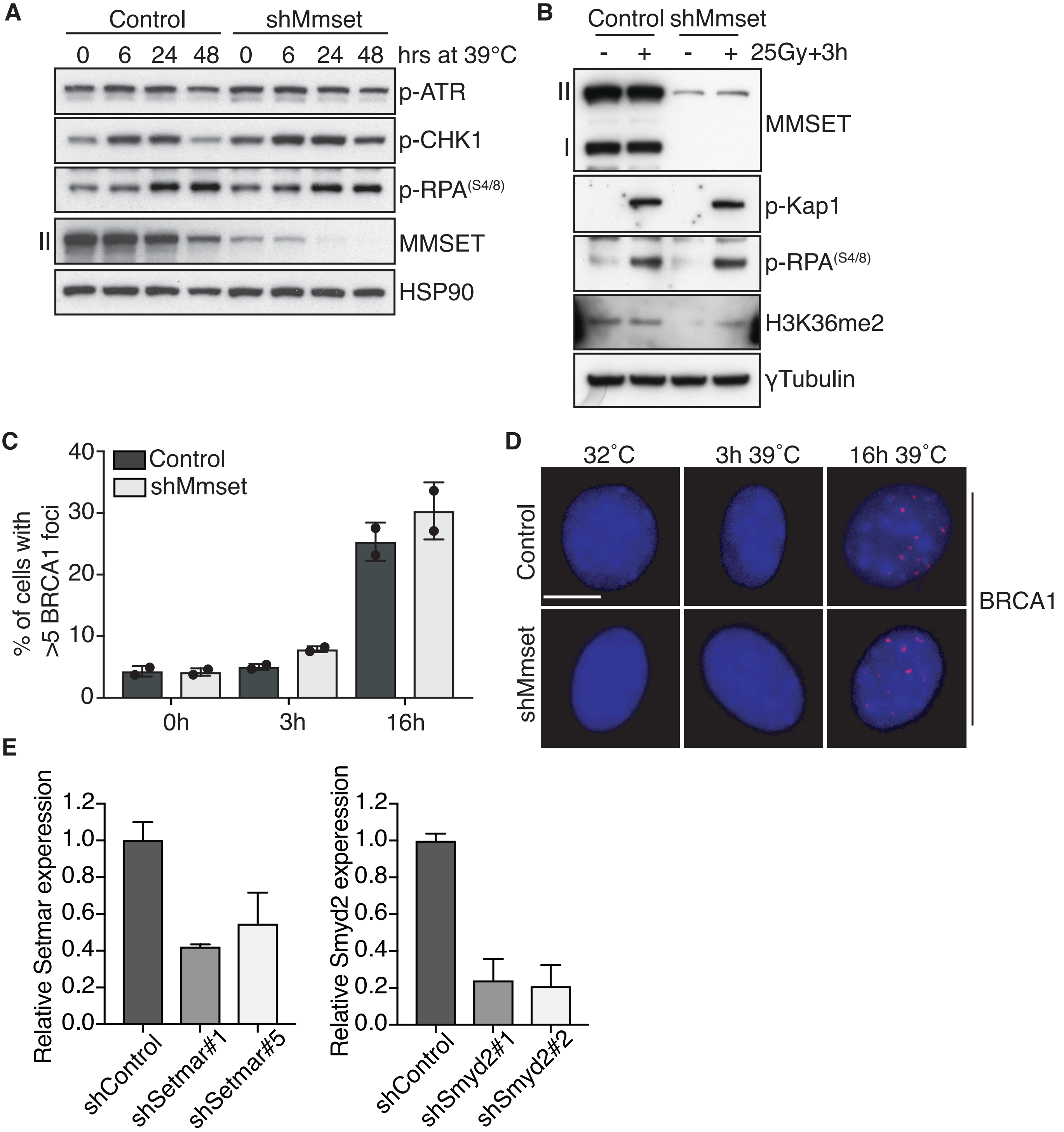

Supplementary figure 5. A. Immunoblot for p-ATR, p-CHK1, p-RPA (Ser4/8) and MMSET in TRF2ts MEFs transduced as indicated and placed at 39°C for indicated times to induce telomere uncapping. HSP90 serves as loading control. (representative of n=2 independent experiments). **B.** Immunoblot for p-RPA (Ser4/8) of MMSET-depleted p53$^{-/-}$ MEFs irradiated with 25Gy and collected 3 h post IR. **C.** Quantification of BRCA1 foci in TRF2ts MEFs placed at the non-permissive temperature of 39°C for 3 h or 16 h or kept at 32°C. In each experiment a minimum of 172 cells were quantified per condition (n=2 independent experiments, mean±s.d.). **D.** Representative images of cells quantified in **C**. Scale bar, 10μm. **E.** Representative qRT-PCR analysis of *Setmar* and *Smyd2* mRNA levels of cells used in Fig. 5B (technical triplicate, mean±s.d.).
