# Supplementary material for: H3K36 dimethylation by MMSET promotes classical non-homologous end-joining at unprotected telomeres
Source: Oncogene. 2020 May 29;39(25):4814–27. doi: 10.1038/s41388-020-1334-0 (PMC7299843; doi:10.1038/s41388-020-1334-0)
Supplement: Supplementary file 2 — Supplementary Materials and Methods [file 41388_2020_1334_MOESM2_ESM.pdf]

Supplementary material and methods

Cell culture and flow cytometry analysis

Phoenix-eco cells, 293T (ATCC) and MEFs were cultured in a humidified, 5% CO_2 incubator in Dulbecco's modified Eagle's Medium (DMEM) supplemented with 100U penicillin, 0.1mg ml^{-1} streptomycin, 2mM L-glutamine and 10% FBS.

For cell cycle analysis, BrdU (B5002, Sigma, 1:1000) was added to cells 1 hour prior to harvest. Cells were collected by trypsinization and spinned down at 1500 rpm for 3 minutes. The cell pellet was resuspended in 1ml DMEM and 2ml of EtOH 100% was added while vortexing. Cells were then incubated with 0,5mg ml^{-1} RNAse A for 30 minutes at 37°C, washed three times with PBS/0,5%Tween-20, resuspended in fresh HCl/H_2O/Triton solution and incubated at room temperature (RT) for 20 minutes. $Na_2B_4O_7$ was added for neutralization and cells were washed with PBS/0,5%Tween. Cells were incubated with anti-BrdU (1:40, DAKO #M074402) in 1%BSA/PBS/0,5%Tween for 30 minutes at RT followed by washing two times with PBS/0,5%Tween. Then, cells were resuspended in 1%BSA/PBS/0,5%Tween containing FITC-conjugated anti-mouse (1:20, DAKO #F0479) for 30 minutes at RT, washed twice with PBS/0,5%Tween and resuspended in PBS containing 20 μg ml^{-1} propidium iodide (PI, #P3566, Invitrogen). Data was acquired on a Calibur (BD) or LSRII SORP (BD) and analyzed with FlowJo software.

To address aneuploidy (>4N) in TRF2ts MEFs, MEFs were seeded at 750,000 cells/10cm dish and placed at the non-permissive temperature (39°C) for 48 hours or kept at 32°C. Cells were harvested as above, treated with 0,5mg ml^{-1} RNAse A for 30 minutes at 37°C, washed three times with PBS/0,5%Tween and resuspended in PBS containing 20 μg ml^{-1} PI. Data was acquired on a Calibur (BD) and analyzed using FlowJo software.

Retroviral and lentiviral transduction

For retroviral production, phoenix-eco producer cells were transfected with plasmid DNA using standard calcium precipitation. Medium was refreshed after 16 and 24 hours. Supernatant containing the viral particles was collected 48 and 62 hours post transfection and filtered through a 0.22μm pore size filter. For infection, viral supernatant was supplemented with 4μg/ml polybrene and added to the cells. pRetrosuper retroviruses encoding the following shRNAs targeting mouse genes were used: *Mmset* shRNA#1: 5'-GGAACAGGAAGAGGAGCAT-3'; *Mmset* shRNA#3: 5'-GAGCTTGGATATTTGAGAA-3'; *Mmset* shRNA#4: 5'-GCACCCACGAAAGCTGAGA-3'; *Trf2* shRNA#2: 5'-GAACAGCTGTGATGATTAA-3'; *Rnf8* shRNA#2: 5'-GCATCAGGCTCTAATGGAA-3' and *Lig4* shRNA#3: 5'-

GGATCAGAGACGAGTTACT-3', as well as a retrovirus containing empty backbone pRetroSuper control. In all experiments *Mmset* shRNA#3 was used to knockdown *Mmset*, unless indicated differently. For lentiviral production, 293T cells were transfected with pLKO-puro shRNA plasmids from the Mission library clones (Sigma) as described before(1). Lentiviral plasmids contain the following target sequences against mouse genes *Parp1* sh#08 (TRCN0000071208, 5'-CCTCTTAGTCTGCTGAGCTTT-4'); *Lig3* sh#1 (TRCN0000070978, 5'- CCAGACTTCAAACGTCTCAAA-3'), *Lig3* sh#3 (TRCN0000070979, 5'-GCAATGAAGAAGTGTCCCAAT-3'), *Setmar sh1* (TRCN0000120847, 5'-CCCACATTCAACTCAGCAATT-3'), *Setmar sh2* (TRCN0000120848, 5'-CCCTACCTACATAGGAAATAT-3'), *Smyd2 sh1* (TRCN0000175920, 5'-GCTATGTTTCAGGTTCACGTT-3'), *Smyd2 sh2* (TRCN0000175755, 5'-GCTCTGACCTTGAGTTCATTT-3'), a scrambled shRNA control (5'-CAACAAGATGAAGAGCACCAA-3') or a luciferase shRNA control (5'-CGCTGAGTACTTCGAAATGTC-3'). For complementation experiments, human *MMSET* expression constructs in a Gateway compatible backbone were obtained and shuttled from pDONR to an Mscv-blas backbone using a Gateway technology cloning kit (Invitrogen). Both the wild-type and the $MMSET^{H1146G}$ mutant constructs were made RNAi resistant by changing the target sequence of *Mmset* shRNA#3 at multiple positions using a QuickChange SiteDirected Mutagenesis kit (Stratagene). The other mutant *MMSET* constructs all lacked the target sequence of the *Mmset* shRNA#3. For ChIP experiments, cells were transduced with a Mscv-blas backbone containing eGFP-MMSET RR or using pLPC GFP-TRF1. A human *FBXL11* expression construct was shuttled from a pDONR to an Mscv-blas backbone using Gateway technology.

Metaphase chromosome analysis

Telomere-fusion assays were performed essentially as described before(1, 2). Briefly, $Trf2^{-/-};p53^{-/-}$;TRF2ts MEFs were placed at the non-permissive temperature (39°C) for 24 hours to induce chromosomal fusions. Alternatively, WT, $53bp1^{-/-}$ or $Rif1^{-/-}$ MEFs were infected with retrovirus containing a shRNA targeting *Trf2* and grown for an additional 5 days after infection and selection. $TRF1^{F/F}TRF2^{F/F}Ku70^{-/-}p53^{-/-}$ Cre-ER^{T2} MEFs were treated with 0.5μM tamoxifen (4-OHT) for indicated timepoints. Olaparib (0.5μM) was added 48 hours prior to metaphase harvesting. Two hours prior to collecting the cells, 10 μg ml^{-1} colcemid (Karyomax) was added to enrich for mitotic cells. Cells were resuspended in 0.075M KCl prewarmed to 37°C and incubated for 7 minutes at 37°C followed by spinning down for 5 minutes at 1000 rpm and fixation in Methanol-Acidic Acid fixative. Telomere FISH was performed on chromosome spreads with a FITC-OO-$(CCCTAA)_3$ peptide nucleic acid custom probe (Biosynthesis) or an Alexa488-C-rich telomere probe (Panagene, Eurogentec). Images were acquired as described before(1, 2).

Quantitative real-time PCR

For RNA extraction, cells were washed with PBS and collected in TRIzol reagent (Ambion) followed by RNA isolation using standard procedures. Subsequently, RNA was reverse transcribed into cDNA using AMV first-strand cDNA synthesis kit for RT-PCR (Roche) or the Thermo Scientific Maxima First Strand cDNA Synthesis Kit. Quantitative real-time PCR (qRT-PCR) was performed in triplicate using Power SYBR green PCR Master Mix (Applied Biosystems) or SensiFAST SYBR Mix (Bioline) on the StepOnePlus real-time PCR system (Thermo Fisher Scientific) or LightCycler 480 II (Roche). Normalization was performed over Hprt. For all qRT-PCR primers see the following table:

Primers	Fw (5'-3')	Rev (5'-3')
Hprt	CTGGTGAAAGGACCTCTCG	TGAAGTACTCATTATAGTCAAGGGCA
Mmset	TGCTTGGGTCCCAGTGGAGCA	AGCTTGTTCTGGCCGTTTTGTCGG
Trf2	GAGAGCCACCTGGATGACAC	TTCTGAGGCTGTCTGCTTGG
Parp1	AGCTGAAGAAAGCGTGTTCCA	GTCCAAGATCGCTGACTCTCCT
Lig3	GGTGGCACGTGGTCTTAGAT	GATCCTCTTCCCGAGAAACC
Rnf8	CCTCTGATGATTTCCCGA	CAGACGTTCTCTGTTCAGCC
Rnf168	AGGAGCCTGAGCACCAGTATCAACA	CAAGGCGGTCCCAGGCTCCTA
Tp53bp1	AGGAATGGAGACAGAAAACCC	CCAGACTTGGTGATGGAAATGAA
Lig4	CATATACCGACCAGTTTCCTGAATC	CCCGAACGATTGTGAATAA
Xrcc4	TTCTCTCATTGGGCGCATCG	TCAGAAACTGTTGCAGTCCAGG
Ctip	GTGCTGGGTCGGAGCAG	TTGACTTGTAAACCTTGCACTTCC
Exo1	TAAACACGTCGAGCCTGTCC	CAGAGCCCAGGAACCTTGTT
Paxip	GCAGCAGCAGCAGCTTTTTG	TGCTCGGGATAGTCCGCAAT
Brca1	GCACAGTGCTGCCCAAGGGT	CATGGTCGCCCGCTGCTGAG
Mad2l2	ACACTCCACTGCGTCAAACC	AAAGACAAACTTCTCCACTGGGC
Rad51	GGCTGGTTTACTGTGTCTTT	TGGAAATACTCCTGATGCAG
Rad50	TGGTAGACTGCCAGCGAGAACT	TCGGATGTGCTCTTGATGGCGA
Artemis	GATGCACTGTGTCCACAAGAT	TTCCTGCCACTCAGGGTGT
Ercc1	GTGGAAAGAGTGATGCGGAAC	GTATGGCCGTCTGAATGGCA
Setmar	GCCTAAACCTTTCCAGTATACTCCT	ATGCAAGCACATCCAGGGAA
Smyd2	GGAGACCAGGTGTTCACCAG	TGAGCTTTCGGACTTCCACC

Immunoblotting and histone extraction

Primary antibodies used were against MMSET (75359, Abcam, 1:1000), p-ATM (Ser1981, 4526S, Cell Signaling 1:1000), p-ATR (2853S, Cell Signaling, 1:1000), CHK2 (1:1000, 611570, BD), p-CHK1 (Ser345,

2348S, Cell Signaling, 1:1000), 53BP1 (A300-272A, Bethyl, 1:10000), γH2AX Ser139 (2577S, Cell Signaling, 1:1000), p-Kap1 (Ser824, A300-767A, Bethyl, 1:1000), LigIV (H300, scbt, 1:300), p-RPA32 (Ser4/8, A300-245A, Bethyl; Ser4/8, NBP1-23017, Novus Biologicals), H3K36me1 (NB21-1251, Novus, 1:1000), H3K36me2 (07-369-1, Millipore, 1:1000; confirmed in dot blot specificity assays by the manufacturer to only recognize H3K36me2 and not H3K36me1, H3K36me3 or unmodified histones), H3K36me3 (Ab9050, Abcam, 1:1000), H4K20me2 (Abcam, ab9052, 1:1000), H4K16-Acetyl (39167, Active Motiv, 1:2000), Acetyl-Histone H3 Lys36 (07-540, Upstate, 1:1000), H3K9me3 (07-442, Upstate, 1:1000), Flag (M2, Sigma, F3165, 1:2500), GFP (a-11122, Invitrogen, 1:500) and RAD51 (sc-8349, SantaCruz, 1:500). Antibodies against γ-Tubulin (clone GTU-88, Sigma, 1:1000), β-Actin (A5316, Sigma, 1:2000), H3 (ab1791, Abcam 1:1000) and Hsp90 (sc-7947, Santa Cruz, 1:1000) were used as loading controls. For detection on the Odyssey Infrared imager (LI-COR), IRDye800CW- and IRDye680-labelled secondary antibodies were used. Horseradish peroxidase (HRP)-conjugated secondary antibodies were used for detection by enhanced chemiluminescence (Supersignal, Thermo Scientific).

For acid extraction of core histones, cells pellets were harvested and three different extraction steps were performed. First, high mobility group (HMG) non-histone chromatin proteins and histone H1 were extracted with 5% perichloric acid (PCA). Then, 0.4N HCl was used to extract core histones from the remaining pellet. Both PCA and HCl extractions were performed in the presence of 10 mM iodoacetamide (Sigma-I1149), 5 mM Sodium Butyrate (Sigma-B5887) and protease inhibitors (Complete EDTA-free Protease Inhibitor Cocktail, Roche). Finally, core histones were precipitated using 25% trichloroacetic acid (TCA) and washed with 100% Aceton/0.006% HCl followed by a 100% Aceton wash. The dried histone pellet was resuspended in 50 mM Tris-HCl pH 7.5-8 and protein concentration was determined by Bradford assay. Samples were supplemented with LDS sample buffer (NuPAGE, NOVEX Life technologies) and ß-mercaptoethanol, loaded onto precast 4-12% SDS-PAGE gels and immunoblotting was performed with standard methods.

Immunofluorescence (IF)

After fixation in PFA cells were permeabilized in 0.5% Triton/PBS for 10 minutes and incubated in blocking solution (0.02% Triton, 5% NGS, 5% FCS in PBS) for 1 hour. Cells were incubated overnight at 4°C in blocking solution with primary antibodies. Next, cells were washed three times with 0.02% Triton/PBS, incubated with Alexa Fluor 488 or 568 goat anti-mouse or anti-rabbit IgG secondary antibodies (Invitrogen) in blocking solution for 1 hour, washed three times with 0.02% Triton/PBS and mounted using DAPI-containing Vectashield (Vector Laboratories).

Primary antibodies used for IF: γH2AX (5636, Millipore, 1:500), p-ATM (Ser1931, 4526S, Cell Signaling), 53BP1 (A300-272A, Bethyl, 1:500), DNA-PKcs phospho S2056 (ab18192, Abcam, 1:500), FK2 (04-263, Millipore, 1:4000), BRCA1 (mBRCA1 antibody generously provided by the Jos Jonkers lab, 1:10).

p-DNA-PKcs foci were captured on a confocal Leica SP5 system using a X63 oil objective with LAS-AF software and analyzed using a customized Macro on ImageJ software. All other DDR-proteins were captured and analyzed using the metafer4/MetaCyte system (MetaSystems) essentially as described before(2)

Chromatin Immunoprecipitation

*$Trf2^{-/-}$;$p53^{-/-}$;*TRF2ts MEFs transduced as indicated were seeded at $2x10^7$ cells per condition, allowed to attach overnight and placed at the non-permissive temperature for 3 hours prior to harvest, or kept at 32°C. Cells were washed with ice-cold PBS and crosslinking was performed using 1% formaldehyde for 15 minutes followed by addition of Glycine to a final concentration of 0.2M for 5 minutes. After washing three times with ice cold PBS, cells were collected and resuspended in 2 ml lysis buffer (50mM TrisHCl pH8, 100mM NaCl, 0,5% SDS) supplemented with 10 mM iodoacetamide (Sigma-I1149), 5 mM Sodium Butyrate (Sigma-B5887), protease inhibitors (Protease Inhibitor Cocktail tablets, Roche) and phosphatase inhibitors (PhosStop, Roche). Lysates were stored at -20°C until further use. For chromatin preparation, 1 ml triton dilution buffer (100mM TrisHCl pH8, 300mM NaCl, 3% triton supplemented with the inhibitors indicated above) was added to the samples and lysates were sonicated on ice with an Active motif EpiShear probe sonicator. Correct fractionation of the chromatin was checked on an agarose gel upon DNA isolation of an aliquot of the lysate. At this point an input sample was taken. ChIP was performed with the following antibodies: H3K36me2 (07-369-1, Millipore), H3 (ab1791, Abcam), GFP (a-11122, Invitrogen, 1:500) or IgG rabbit isotype control (ab171870, Abcam). Antibodies were pre-coupled overnight to protein A and protein G magnetic beads (Dynabeads, Invitrogen) prior to incubation with the lysate. After overnight incubation at 4°C, immunoprecipitated protein-DNA complexes were washed twice with wash buffer A (20mM TrisHCl pH8, 300 mM NaCl, 0.1% SDS, 1% triton), once with wash buffer C (10mM TrisHCl pH8, 0.25M LiCl, 1% NP-40, 1% Na-Deoxycholate, 1mM EDTA) and subsequently with TE+NaCl (10mM TrisHCl pH8, 1mM EDTA, 100mM NaCl). Samples were eluted from the beads by addition of 300 μl elution buffer (1% SDS, 0.1M $NaHCO_3$) while shaking at RT for 30 minutes. A second elution was done by adding 200 μl elution buffer for 10 minutes while shaking. The input samples were supplemented with 450 μl elution buffer and 20 μl NaCl (5M) was added to both input and elution samples prior to incubation at 65°C for 6 hours to reverse the crosslinking. After

centrifugation (2min, 14000 rpm), samples were supplemented with 0.5M EDTA, 1M TrisHCl pH7, 40 μg of RNAse and incubated at 37°C for 30 minutes. Then, samples were incubated with 60 μg Proteinase K and incubated for 1 hour at 37°C. DNA was recovered by phenol-chloroform extraction and ethanol precipitation.

Subtelomeric sequences of chromosome 1, 16 and 19 were detected using the following primer pairs described before(4, 5): chromosome 1 fw 5'-TTAGGACTTCTGGCTTCGGTAG-3' and rev 5'-AGCTGTGGCAGGCATCGTGGC-3'; chromosome 16 fw 5'-CACCTTTAACACCCCTTCCA-3' and rev 5'-CAGAGCACATGGAGTGGAGA-3' and chromosome 19 fw 5'-TTTTTCTTGCTGGACGAGGT-3' and rev 5'-ACAAAAGCAGAGGCCAGAAA-3'. Primers targeting the GAPDH coding region (fw 5'-GGAGAGTGTTTCCTCGTCCC-3' and rev 5'-ACTGTGCCGTTGAATTTGCC-3') were used as a positive control for the H3K36me2 pulldown. For each sample qRT-PCR was carried out in triplicate using Power SYBR green PCR Master Mix (Applied Biosystems) on a StepOnePlus real-time PCR system (Thermo Fisher Scientific). Values are calculated by the cycle difference between the immunoprecipitated sample and the corresponding input sample and normalized to IgG sample to determine specificity of the pulldown or normalized to control sample. ChIP values of shRNA-transduced MEFs are represented relative to control-transduced MEFs.

For telomere dot blots, ChIP samples were loaded onto a Hybond-N^+ membrane (Amersham Hybond-N^+, GE Healthcare) using a 96-well dot blot manifold (BRL Hybri Dot manifold 1050MM). The input samples were diluted as indicated. After sample addition, the membrane was denatured for 10 minutes using 1.5M NaCl/0.5N NaOH followed by neutralization with 1M NaCl/0.5M Tris-HCl pH7 and crosslinking (Stratagene UV crosslinker). After rinsing the membrane in 2xSSC, the membrane was pre-hybridized in Church Mix (0.5M NaPi pH7.2, 1%BSA, 7%SDS and 1mM EDTA) at 65°C for 30 minutes and hybridized overnight at 65°C in Church Mix with a ^{32}P-labeled telomere repeat-probe generated from an 800-bp telomeric DNA insert (pSP73Sty11) using Klenow polymerase, $(CCCTAA)_3$ primer and ^{32}P-alpha-dCTP. Next, the membrane was washed three times at RT in 2xSSC. Signal was obtained on a phosphorimager and analyzed with AIDA Image Analyzer software.

References

1. Peuscher MH, Jacobs JJ. DNA-damage response and repair activities at uncapped telomeres depend on RNF8. Nature cell biology. 2011;13(9):1139-45.

2. Boersma V, Moatti N, Segura-Bayona S, Peuscher MH, van der Torre J, Wevers BA, et al. MAD2L2 controls DNA repair at telomeres and DNA breaks by inhibiting 5' end resection. Nature. 2015;521(7553):537-40.

3. Konishi A, de Lange T. Cell cycle control of telomere protection and NHEJ revealed by a ts mutation in the DNA-binding domain of TRF2. Genes & development. 2008;22(9):1221-30.

4. Gonzalo S, Jaco I, Fraga MF, Chen T, Li E, Esteller M, et al. DNA methyltransferases control telomere length and telomere recombination in mammalian cells. Nature cell biology. 2006;8(4):416-24.

5. Benetti R, Garcia-Cao M, Blasco MA. Telomere length regulates the epigenetic status of mammalian telomeres and subtelomeres. Nature genetics. 2007;39(2):243-50.

shRNAs Methyltransferase Screen

Pool	Target	shRNAs			
**Pool 2**	***Ash1l***	TGGGATTGGTTAATAAGGA	GGACATTGGAAAGAAACTA	CCTCGGTGGACTAAAGTGG	TGAAGGAACATGCATTCAG
	Smyd1	GAGGCAGGAGAAGCTCCAT	CCACGTGGAGCACTTTGGG	CCTGGGCCTGGTGAACCAT	TGGCAATCATGAGGCAGTG
	Kmt5b	TGACCATCAGCAGAATCAA	CCAGTTTGGTTCTTGATCC	GTTCGGCAATACAACCCAC	TCCACTGTCAGTTGCAAAC
	Dot1l	GTGGATCACCAGCTGAAGG	CCAGTACCTGCAGTTCCTG	GTGGATGAAATGGTATGGA	GATTGATGAGATCAAGATG
	Ezh1	TGACTGGGCCTCCAGTTCT	GCAGGTCTTTCAGTTTGCA	GAAGAGATGATCCCTGGAT	GCTGCCAACAGATGAGCTC
**Pool 3**	***Ezh2***	TCATGGGCCAGACTGGGAA	TGTGGAGTGGAGTGGTGCT	GCCATTTCCTCAATGTTTC	CATGTAGACAGGTGTATGA
	Setd5	CGCTGTGGAGACAGCCCGA	GGTTATTAGACTTCATCGG	GCAGGACAACATATCAGGT	GCTGGGATGAGGAGCTTTC
	Setd2	GCGAATGCAGTGTGAGTGT	TGCAGTGTGAGTGTACACC	GAAGGATTCAGTGGATGGA	CCAGAAGCTTCAGGAAGAG
	Smyd2	TGTCATTGTGACCTACAAA	GGGACCCTGGCAGAAGTCA	GCCATCCGAGACATGGTCA	CTGCTGGAGATCTGCGAGC
	Smyd5	GGCAGAGGAGAATGCCCAG	GATGGCGGCCTCCATGTGC	CTGTGAAGGATCTGGCCTC	TGCCTGGCAGAGGCTGATG
**Pool 4**	***Nsd1***	TATACAGAAGAATATGATC	TTGTTTGCACTTCCTTGAG	GATTCTCCATCTTGTTACA	GGACAGCCCTTTCGGTAAT
	Prdm1	CGGGATGAACATCTACTTC	CTTCTTGTGTGGTATTGTC	GAGAGAGTACAGCGTGAAA	GGTCCACCTGAGAGTGCAC
	Prdm10	TGCAGCTCTTCAGCATCTG	GCAGGAACTGAAGGTGTGG	GTTCTTCGAGAGCAAGAGA	GTGCCGCCTGTGCATGATG
	Prdm12	GCCCTTCGTGTGCCGCTTC	CAACAACCTCATGTGGGAG	GCATGAGGACTTCCACCCG	CTCGCGCAGCAACCTGCGC
	Prdm13	GATCCACATGCGGACGCAC	TACGCCCTACCGCTGCGAG	TTGGTCAAGTTGCAATTAT	CAGACTCTGGAAGCTATTG
**Pool 5**	***Prdm16***	GGCGAGGGCGAGGAAGCTA	GTGACGGTGACGTTGTAAA	GCATATCCACAGCACGGTG	GTCCTACACGCAGTTCTCC
	Prdm17	GGCATTCCTACAGCTGTGC	CTGCGGCGCCATCTCATCT	GCCAGGAGAAGTGCTTTGT	GCTGGAGGAAGAAGTAGC
	Prdm2	GAATGGCCCTTCAAATGTG	GTGACAAGAAGAGGTACAC	GAGGCCAATGGTGATGTAT	GCTCTTCATCCTTGAGGAA
	Prdm4	TACAGCATTATCTATCACC	TGGAAATGTTGGATTAGAA	TCCCAAATGGCTCCAGAAG	CACACATGGTGCCCAAAGT
	Prdm5	GATTCATGAGATATTTGAT	GATGCACATTCGTACTCAC	TGCCTGAAGACTTGGATGA	TGACTACCTCAAAGTGCAC
	Prdm6	GTCATGATTAAATGTCACG	TCAATCGAAGTGGATTAAC	GCCCTTCAAGTGCGAGAGG	TGTCACGGACACTTAAGCA
**Pool 6**	***Prdm7***	CTGCTATGAGTATGTGGAT	CTGGATGAGGTATGTGAAC	CTGTGCCCGGGATGATGAA	GAGCAGAACCTGGTAGCCT
	Prdm8	TGGTTCCTCAGAAGGTCTC	CGTTTCCAGTTTGAGTTCC	GCGCAGCGCCTTCGTGGAG	GAGAACAGTACCTTACATC
	Prdm9	CTGCTATGAGTATGTGGAT	CTGTGCCCGGGATGATGAA	TACGGCCAGGAACTGGGCA	CTGGATGAGGTATGTGAAC

	REIIBP	CCACTGAAGAAGCGAAATC	GTTCATCTCCTTCTGCATC	CTACAGATGGTGGCCGGCA	GCACGAGATTGGAGAATTC
	Kmt5a	CCATTAGCTGGAATCTACA	CTGCACGACATCGACGGCG	GGTGGACTTGAACAGATGG	TATCTGTTACAGGTTTCCA
	Setd7	GGGCACCTGGACGATGACG	TTACACACCAAGAGGTTGA	GTACTGTGCCTCCTTGGGA	GTGGAGCTGAAGGCCTTCC
**Pool 7**	***Setdb1***	GATGATGATGTCCTCAGTA	GCTATGGCTGCCTTAAGAA	GTTCATGGATGCTGTCAAC	CCATATTGCCTATGATTAC
	Unc	GCTGGAGCTGCTGGTGGGC	CCATGACTGCAAACCCAAC	TGAGAAGCTGGAGCTGCTG	GAATGAGCACTGTGAATGC
	Mmset	GGAACAGGAAGAGGAGCAT	CGTTGGTGATTTGGTGTGG	GAGCTTGGATATTTGAGAA	GCACCCACGAAAGCTGAGA
	Whsc1l1	TTCAGCAACCACCTCAACT	CTATCCAGAATGGCAGGGA	TGGCAGGGAATTGTTTGAG	GGTGCCAGCGAGATTTCAG
	Setmar	CTCCATGTGTCCCTGGCAC	GGAAAGTACGCCAAGCCAG	CAAGATGTAGCGTGCGGCC	GATTTGTCTGTGAGTATGC
**Pool 8**	***Smyd3***	GCTGATGCGTTGTTCTCAA	GCAGCTGAGGGACCAGTAC	GTGATGAAAGTTGGCAAGC	GCCATGAAGAACCTGAGGC
	Prdm11	GTCAGCTGGCTTCTTCTCA	GTGTCCTCCTGTGTGGGTG	CTGAAGGGAAAGCGTGACC	GACCTCAGCCAACCCGTCT
	Prdm15	CTCCATCCTGACTGTGACC	CCCTCAGTCAGTGGCCCAC	GCAGTGGACTTGTTCCGTG	GCCACAAACCCTCAGTCAG
	Setd1b	GAGCCACATTCACGACTGG	GATGGTAGAGGTGGTGGCC	GCCGACAGCGATGGCCAGA	CACCTGGGCATTGCCAAGG
	Kmt2b	CTTCCTGGCCTCCCAGCAC	GAAGAGGATGAGGTGCAGC	GATGCCAGCAACAAGCTGC	CTGTGGCGCCAAGCGCTGC
	Kmt2a	GTCTCGGCCGCCATCGGCA	ATGAAGAAGTCAGAGTGCG	GTCTAAGTTTAAGACAGGG	GACAGTTGTCAGACAAAGC

shRNAs H3K36me2-specific histone methyl transferases

Nsd1 – Pool I	***Nsd1 – Pool II***	***Whsc1l1***	***Ash1l***
GATACAGGTGACTGGTTGCAA	CCACTGTTCTTCTTTCCTTAT	CTCCTCAAGAACCTATACTTT	GCTGCCACATTTGGCTCTAAA
CAGAGAGGCATCGTGATACTT	GCAGCAAGATTTGAAGAGTTA		CCTCCTACTTTGTTGCCAAAT
CAATCAGGATTCAGATGTGCA	GCTCGTTAAGACACCAGGAAA		CCCATTCATGTTGGAAAGTAT
CTAGGTTCAGAAACACTGTCA	GCTAACAGATTTAGGGAGAAA		GCCTCACAGAAAGGAACCATT
	CCAGATTTCTTTCTCCGCCTT		GCTGGTCATTTATTGCTCAAT

Setmar	***Smyd2***	***Setd3***
CCCACATTCAACTCAGCAATT	GCTATGTTTCAGGTTCACGTT	CGAAAGTTACTAATGACTGTT
CCCTACCTACATAGGAAATAT	GCTCTGACCTTGAGTTCATTT	GCTGGAGATCAGATTTACATT
CGACCCTACCTACATAGGAAA	GCAGAAGATCATTAAGCCGTA	GAGGTCAAACTTTGGACATTT
CCAAGTAAGTAGGAAAGACAA	CCAGGTGAACTGTAATGGCTT	CATCACCATGTTCCTTGTTAA
CGCCATGAGAATAACTACGAT	CAACGAGAAGAAAGATCTCAT	
